# Artificial Intelligence–Enabled ECG for Diastolic Dysfunction in Congenital Heart Disease: A Novel Risk Stratification Tool

**DOI:** 10.1016/j.jacadv.2025.102413

**Published:** 2025-12-16

**Authors:** Donnchadh O’Sullivan, Malini Madhavan, Sahar Samimi, Scott Anjewierdan, William R. Miranda, Zachi I. Attia, Heidi M. Connolly, Katia Bravo-Jaimes, C. Charles Jain, Paul A. Friedman, C. Alexander Egbe, Francisco Lopez-Jimenez, Jae K. Oh, Luke J. Burchill

**Affiliations:** a Department of Pediatric Cardiology, Texas Children’s Hospital, Houston, Texas, USA; b Department of Cardiovascular Medicine, Mayo Clinic, Rochester, Minnesota, USA; c Baylor College of Medicine, Houston, Texas, USA

**Keywords:** adult congenital heart disease, artificial intelligence, diastolic dysfunction, invasive hemodynamics

## Abstract

**Background:**

Echocardiography-based assessment of diastolic function in adult congenital heart disease (ACHD) is challenging owing to complex anatomy and heterogenous physiology.

**Objectives:**

The objectives of the study was to evaluate an artificial intelligence (AI)–enabled electrocardiogram (ECG) (AI-ECG) model for grading diastolic dysfunction in patients with ACHD and assess its correlation with echocardiographic, invasive hemodynamic, and clinical outcomes.

**Methods:**

In this single-center retrospective study, we analyzed 6,741 patients from the Mayo Clinic ACHD Registry (median age 37 years, 49% female) followed from 2000 to 2023. The median follow-up was 10 (5-15) years. Using a validated deep neural network (trained on 98,736 ECG-echocardiogram pairs), we assigned an AI-ECG diastolic grade (0-3) to the earliest ECG within 12 months of the index visit. We evaluated associations with echocardiography, hemodynamics, and mortality using nonparametric tests, correlation, Kaplan-Meier curves, Cox regression, and model performance for detecting elevated pulmonary artery wedge pressure (PAWP).

**Results:**

The AI-ECG classified diastolic function as grade 0 in 65.8%, grade 1 in 4.0%, grade 2 in 19.7%, and grade 3 in 10.5%. Higher grades were associated with older age, greater CHD complexity, and more comorbidities including heart failure (6.7% vs 24.8%), diabetes, and cirrhosis (all *P* < 0.001). N-terminal pro–B-type natriuretic peptide rose with each grade (129 [60-304] to 763 [311-1,915] pg/mL; *P* < 0.001). AI-ECG pressure estimates correlated with left atrial strain (ρ = −0.52) and right ventricular strain (ρ = −0.50). Invasive hemodynamics followed similar patterns; right atrial pressure rose from 8 (6-11) to 13 (10-18) mm Hg, and wedge pressure from 11 (8-14) to 16 (12-21) mm Hg (*P* < 0.001). Survival differed by grade (log-rank *P* < 0.0001); grades 2 and 3 independently predicted mortality (HR: 1.38; 95% CI: 1.09-1.75; HR: 1.63; 95% CI: 1.27-2.08). The model discriminated pulmonary artery wedge pressure ≥20 mm Hg with an area under the receiver operating characteristic curve of 0.74 (95% CI: 0.70-0.78).

**Conclusions:**

AI-ECG diastolic grading correlates with echocardiographic and invasive measures of cardiac filling pressures and independently predicts mortality in ACHD. These findings support its utility as a scalable, noninvasive risk stratification tool.

Diastolic dysfunction is a driver of clinical outcomes in adults with congenital heart disease (CHD), contributing to heart failure progression and mortality across diverse anatomical subtypes.^1^, ^2^, ^3^ The complex interplay of congenital defects, surgical intervention, and acquired comorbidities creates unique diastolic pathophysiology in adult CHD (ACHD) characterized by ventriculoventricular interaction, altered loading conditions, and adaptive myocardial changes.^4^, ^5^, ^6^ Traditional echocardiographic assessment, although foundational, can be limited in ACHD due to anatomical variability and postsurgical geometry,^7^, ^8^, ^9^ frequently leading to indeterminate classification of diastolic function. Contemporary echocardiographic algorithms for grading diastolic dysfunction, derived in structurally normal biventricular hearts, are not recommended for complex congenital anatomy.^10^ In particular, noninvasive assessment of filling pressure in patients with systemic right ventricles or single-ventricle physiology is hindered by the absence of reference values and by hemodynamic conditions that violate the assumptions of standard Doppler indices.^11^ Across the congenital spectrum, pressure-overload lesions, volume-overload states, mixed lesions, ventricular compliance, and the diastolic pressure–volume relationship are altered in lesion-specific ways that standard algorithms cannot capture.^12^ Overcoming these limitations, invasively measured pulmonary artery (PA) wedge pressure (PAWP) is an objective surrogate of elevated filling pressure against which to benchmark the performance of any noninvasive marker of diastolic function. Invasive hemodynamic evaluation remains the diagnostic gold standard, but access, cost, and procedural risks limit routine application.^13^

Recent advances in artificial intelligence (AI)–enabled electrocardiography (ECG) (AI-ECG) have demonstrated remarkable capabilities in detecting subclinical cardiac dysfunction, including left ventricular (LV) systolic impairment and diastolic abnormalities in general populations.^14^, ^15^, ^16^ Our institution previously developed a deep neural network of >274,700 ECG-echocardiogram pairs (trained, validated, and tested in 98,736, 21,963, and 98,763 patients), achieving an area under the receiver operating characteristic curve (AUC) of 0.911 for detecting elevated filling pressures individuals without CHD.^17^ To date, this model has not been applied to the patient with ACHD population.

The primary aim of the current study is to validate an AI-ECG model of diastolic dysfunction and its influence on mortality risk in a large patient with ACHD cohort. The secondary aim is to correlate the AI-ECG diastolic dysfunction model with invasive hemodynamic markers across anatomical subtypes. We hypothesize that the AI-ECG model detects elevated intracardiac filling pressures in patients with ACHD, correlates with invasive hemodynamic parameters, and independently predicts mortality risk across congenital subtypes. By addressing the critical gap in personalized noninvasive diastolic assessment for CHD, this work advances risk stratification paradigms in this growing patient population.

## Methods

We conducted a single-center, retrospective cohort study of patients with ACHD identified via the Mayo Clinic ACHD Registry^18^ (Supplemental Appendix 1), which includes patients receiving ambulatory or hospital care at the Mayo Clinic, with follow-up extending from the initial clinic visit until death or last known contact (January 6, 2000-December 26, 2023). The study was approved by the Mayo Clinic Institutional Review Board (IRB #20-007695) with a waiver of informed consent and was conducted in accordance with the STROBE (Strengthening the Reporting of Observational Studies in Epidemiology) guidelines (Supplemental Appendix 2) for observational cohort studies. Eligible patients had at least one standard 12-lead ECG using a GE-Marquette machine which was stored in the GE-MUSE system (Marquette).

### AI-ECG diastolic dysfunction model

The AI-enabled ECG algorithm was previously derived and validated at Mayo Clinic using 4 parameters of diastolic function: early diastolic mitral (or left atrioventricular) annulus velocity (e′), ratio between early diastolic mitral (or left atrioventricular) valve inflow velocity (E) and e′ (E/e′), left atrial (LA) volume index, and tricuspid (or right atrioventricular) valve regurgitation (TR) velocity.^19^ The model was shown to predict diastolic dysfunction severity (grades 0-3) based on deep neural network analyses of standard 12-lead ECG waveforms.^14^ Normal and grade 1 were considered normal filling pressure, and grades 2 and 3 were considered increased filling pressure.

This AI-ECG diastolic dysfunction model was developed and validated using echocardiographic indices of diastolic function.^17^ In the present study, in addition to assessing the echocardiographic indices, we evaluated the model’s performance against invasive hemodynamic measurements obtained during cardiac catheterization. Patients were stratified into 4 groups based on the AI-ECG predictions: grade 0, grade 1, grade 2, and grade 3. Invasive parameters included mean right atrial (RA) pressure; PAWP; PA systolic, diastolic, and mean pressures; pulmonary vascular resistance (PVR); and CI. For this manuscript, “left heart pressures” refers to pressures obtained in the atrium receiving pulmonary venous return. “Right heart pressures” refers to pressures obtained in the atrium receiving systemic venous return. For each patient, the model’s continuous estimated filling pressure was correlated with these invasive measurements to assess its predictive accuracy relative to the gold standard catheterization data.

### Covariates

Each comorbidity was defined as present or absent based on clinical diagnoses documented at the index visit, encompassing both CHD group classification (eg, right/left heart defects, shunt lesions, cyanotic heart disease, systemic right ventricle, and Fontan circulation) and a range of acquired conditions, including heart failure (defined as NYHA functional class II to IV and concurrent loop diuretic use), various arrhythmias (atrial fibrillation, flutter/tachycardia, and ventricular arrhythmias), hypertension, hyperlipidemia, diabetes mellitus, obesity, coronary artery disease, thrombotic events (venous thrombosis, pulmonary embolism), stroke, obstructive sleep apnea, chronic kidney disease, infective endocarditis, and cirrhosis. Clinical heart failure was defined as the simultaneous presence of NYHA functional class II, III, or IV symptoms and concurrent prescription of loop diuretic agents at the index visit. ACHD severity was classified according to defect complexity (mild, moderate, and severe) in keeping with the Bethesda criteria.^20^ The CHD group classification encompassed a diverse range of congenital heart defects, each categorized based on anatomical and physiological characteristics (Supplemental Table 1).

Total pulmonary resistance index was computed as the mean pulmonary arterial pressure (PAP) divided by the cardiac index (CI) (mean PAP/CI); the PVR index was derived by subtracting the PAWP from the mean PAP and dividing the result by the CI ([mean PAP – PAWP]/CI); and the systemic vascular resistance index was calculated as the difference between the mean aortic pressure and RA pressure divided by the CI (mean aortic pressure − RA [superior vena cava] pressure)/CI). N-terminal pro–B-type natriuretic peptide (NT-proBNP) values were log-transformed to account for right-skewed distribution. The transformed variable was defined as log(NT-proBNP) = log1p(NT-proBNP), which preserves interpretability for low values while stabilizing variance.

### Echocardiographic indices

Transthoracic echocardiograms performed as part of routine clinical care were retrospectively reviewed for all patients. Measurements followed the American Society of Echocardiography guidelines, and echocardiographic parameters were extracted from finalized reports in the electronic health record. The first echo within 12 months of the index visit was used. AI-ECG diastolic dysfunction grades (0-3) were compared among echocardiographic measurements and additionally the AI-ECG model’s continuous estimated filling pressure was correlated with each echocardiographic parameters.

### Mortality

All-cause mortality, ascertained by review of medical records and the Accurint mortality database, served as the primary endpoint. Mortality was ascertained using the Mayo Clinic ACHD registry and Accurint database, a commonly used national data set that provides mortality data based on >33 billion records from >8,800 different data sources.^21^

### Statistical analysis

AI-ECG diastolic grades (0-3) were compared to identify group differences in patient with ACHD characteristics. Continuous variables are presented as median (IQR) and compared across AI-ECG diastolic grades using the Kruskal-Wallis test; when the omnibus test was significant, Dunn’s pairwise tests with Holm adjustment were used. Categorical variables were compared using chi-square tests, and for variables with significant global tests, we additionally conducted pairwise grade-to-grade 2 × 2 comparisons with Holm-adjusted *P* values and 2-sided α = 0.05. We assessed monotonic associations using Spearman rank correlation and computed 95% CIs for ρ via bias-corrected and accelerated bootstrap (2,000 resamples), overall and within CHD subgroups. Variables chosen for subsequent multivariate models included an a priori, clinically prespecified covariate set that was deemed clinically relevant, while also controlling for collinearity. Cox proportional hazards models were then fitted to estimate HRs with 95% CIs. In the multivariable Cox model, we assessed proportional hazards using scaled Schoenfeld residuals (global test *P* = 0.604) with visual inspection for each covariate; no violations were detected for the model overall or for individual covariates. Age at first visit and year of birth were checked for collinearity to avoid redundancy, and model discrimination was summarized with the C-statistic. Kaplan-Meier survival curves were generated to compare event-free survival across AI-ECG diastolic grades; survival curves were truncated at the last observed event in each stratum, with numbers at risk displayed below the plot. All statistical analyses were performed using R (version 4.1.0, R Foundation for Statistical Computing). Throughout all analyses, a 2-sided *P* < 0.05 was considered statistically significant. Results are reported as point estimates (HR) with 95% CI.

### AI-ECG filling pressure validation

Model output probability for elevated filling pressure was defined as the sum of the soft-max probabilities for grades 2 and 3, *P*(grade 2) + *P*(grade 3). We evaluated the AI-ECG probability against invasive PAWP ≥20 mm Hg using receiver operating characteristic analysis, reporting the AUC with DeLong 95% CI and plotting a 2,000-bootstrap sensitivity confidence band across specificities. The optimal probability threshold was identified by the Youden index, with corresponding sensitivity, specificity, positive predictive value, and negative predictive value reported. Model calibration was assessed both overall and within each CHD anatomical group. We used logistic recalibration by fitting a logistic regression model with the observed binary outcome (PAWP ≥20 mm Hg) as the dependent variable and the logit of the original prediction as the independent variable; we report the calibration intercept and slope with 95% Wald CIs. Brier scores were computed before and after recalibration with bootstrap 95% CIs (2,000 resamples). To explore heterogeneity in calibration, we repeated this recalibration procedure within each CHD anatomical group. A decile-based and locally estimated scatterplot smoothing–smoothed calibration plot was also generated for the full cohort after recalibration.

## Results

### Cohort characteristics

Among 7,193 patients with CHD whom had echocardiographic data available from initial visit, 6,741 (93.7%) had an ECG within 1 year of their index visit. The median follow-up was 10 [5-15] years. Using the earliest ECG available per patient, within 1 year of the echocardiogram, the AI-ECG algorithm classified diastolic function as grade 0 in 4,432 patients (65.7%), grade 1 in 290 (4.3%), grade 2 in 1,303 (19.3%), and grade 3 in 716 (10.6%). Higher AI-ECG grades were associated with clear differences in demographics, CHD complexity, and comorbidity profiles (Table 1). Patients with higher AI-ECG grades were older than those in grade 0 (median age [IQR]: grade 0, 34 [24-47] years; grade 3, 44 [31-58]; *P* < 0.001) (post hoc in Supplemental Table 2A). The proportion of complex CHD rose from 422/4,432 (9.5%) in grade 0 to 254/716 (35.5%) in grade 3 (*P* < 0.001). Heart failure increased from 301/4,432 (6.8%) to 184/716 (25.7%) (∼3.8×; *P* < 0.001), and atrial as well as ventricular arrhythmias became progressively more common across grades (all *P* < 0.001). In grade 0, systemic hypertension affected 936/4,432 (21.1%) vs 188/716 (26.3%), diabetes 254/4,432 (5.7%) vs 76/716 (10.6%), chronic kidney disease 128/4,432 (2.9%) vs 85/716 (11.9%), cirrhosis 34/4,432 (0.8%) vs 51/716 (7.1%), and infective endocarditis 79/4,432 (1.8%) vs 44/716 (6.1%) (all *P* < 0.001), for grade 0 and grade 3, respectively. For categorical variables with significant global tests, post hoc pairwise comparisons with Holm adjustment were performed and were concordant with the global findings (Supplemental Table 2B).

**Table 1:** Basic Characteristics of Study Cohort by AI-ECG Diastolic Grade

	AI-ECG Diastolic Grade 0 (n = 4,432)	AI-ECG Diastolic Grade 1 (n = 290)	AI-ECG Diastolic Grade 2 (n = 1,303)	AI-ECG Diastolic Grade 3 (n = 716)	*P* Value
Age at first visit, y	34 (24, 47)	48 (33, 62)	40 (29, 55)	44 (31, 58)	<0.001
Male	2,110 (47.6%)	138 (47.6%)	659 (50.6%)	376 (52.5%)	0.041
CHD groups					<0.001
RHD/conotruncal lesions	1705 (38.5%)	79 (27.2%)	286 (21.9%)	193 (27.0%)	
Left heart disease	973 (22.0%)	56 (19.3%)	287 (22.0%)	114 (15.9%)	
Shunt lesion	1,421 (32.1%)	105 (36.2%)	288 (22.1%)	166 (23.2%)	
Cyanotic heart disease	53 (1.2%)	8 (2.8%)	58 (4.5%)	30 (4.2%)	
Other CHD	87 (2.0%)	7 (2.4%)	17 (1.3%)	9 (1.3%)	
Systemic right ventricle	78 (1.8%)	16 (5.5%)	224 (17.2%)	104 (14.5%)	
Fontan palliation	115 (2.6%)	19 (6.6%)	143 (11.0%)	100 (14.0%)	
CHD severity					<0.001
Mild severity	1,258 (28.4%)	65 (22.4%)	192 (14.7%)	116 (16.2%)	
Moderate severity	2,752 (62.1%)	177 (61.0%)	644 (49.4%)	346 (48.3%)	
Complex severity	422 (9.5%)	48 (16.6%)	467 (35.8%)	254 (35.5%)	
Heart failure	301 (6.8%)	30 (10.3%)	211 (16.2%)	184 (25.7%)	<0.001
Atrial arrhythmia	802 (18.1%)	47 (16.2%)	435 (33.4%)	398 (55.6%)	<0.001
Atrial fibrillation	503 (11.3%)	28 (9.7%)	295 (22.6%)	311 (43.4%)	<0.001
Atrial flutter/tachycardia	426 (9.6%)	26 (9.0%)	218 (16.7%)	190 (26.5%)	<0.001
Ventricular arrhythmia	198 (4.5%)	12 (4.1%)	105 (8.1%)	86 (12.0%)	<0.001
Sustained VT	63 (1.4%)	2 (0.7%)	29 (2.2%)	41 (5.7%)	<0.001
Nonsustained VT	131 (3.0%)	9 (3.1%)	68 (5.2%)	55 (7.7%)	<0.001
VF/cardiac arrest	30 (0.7%)	2 (0.7%)	27 (2.1%)	23 (3.2%)	<0.001
Hypertension	936 (21.1%)	91 (31.4%)	340 (26.1%)	188 (26.3%)	<0.001
Diabetes	254 (5.7%)	28 (9.7%)	113 (8.7%)	76 (10.6%)	<0.001
Smoking history	697 (15.7%)	44 (15.2%)	218 (16.7%)	126 (17.6%)	0.529
Obesity	1,006 (22.7%)	95 (32.8%)	293 (22.5%)	130 (18.2%)	<0.001
Coronary artery disease	157 (3.5%)	24 (8.3%)	97 (7.4%)	50 (7.0%)	<0.001
Venous thrombosis	52 (1.2%)	7 (2.4%)	33 (2.5%)	24 (3.4%)	<0.001
Left heart thrombus	11 (0.2%)	2 (0.7%)	15 (1.2%)	11 (1.5%)	<0.001
Stroke	154 (3.5%)	26 (9.0%)	81 (6.2%)	53 (7.4%)	<0.001
Pulmonary embolism	39 (0.9%)	4 (1.4%)	18 (1.4%)	9 (1.3%)	0.279
OSA	487 (11.0%)	49 (16.9%)	187 (14.4%)	106 (14.8%)	<0.001
Chronic kidney disease	128 (2.9%)	17 (5.9%)	97 (7.4%)	85 (11.9%)	<0.001
Infective endocarditis	79 (1.8%)	9 (3.1%)	52 (4.0%)	44 (6.1%)	<0.001
Protein losing enteropathy	11 (0.2%)	0 (0.0%)	14 (1.1%)	15 (2.1%)	<0.001
Cirrhosis	34 (0.8%)	6 (2.1%)	54 (4.1%)	51 (7.1%)	<0.001

Values are median (IQR) and compared using the Kruskal-Wallis test, or n (%) analyzed using the chi-square or Fisher exact test, unless otherwise indicated. This table summarizes the clinical characteristics of the study cohort AI-ECG diastolic grade.

AI-ECG = artificial intelligence–enabled electrocardiogram; CHD = congenital heart disease; OSA = obstructive sleep apnea; RHD = right heart/conotruncal lesions; VF = ventricular fibrillation; VT = ventricular tachycardia.

**Figure 1:**
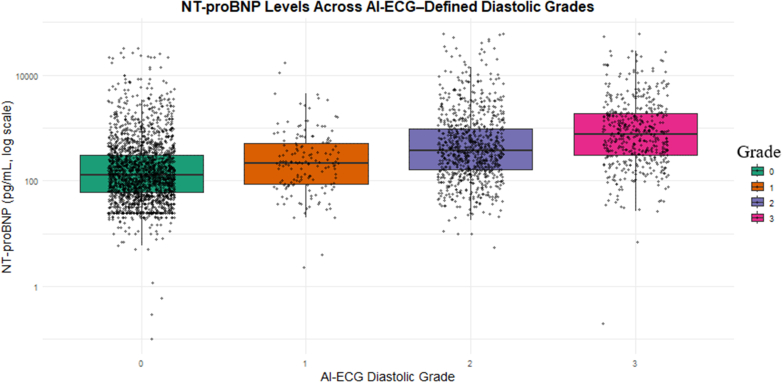
**Boxplot Illustrating the Distribution of N-Terminal Pro–B-Type Natriuretic Peptide Concentrations Across Artificial Intelligence–Enabled Electrocardiogram–Derived Diastolic Grades (Grades 0-3)** Median NT-proBNP levels increased with higher diastolic grade: 129 pg/mL (IQR: 60-304) in grade 0 (n = 2,112), 216 pg/mL (IQR: 87-520) in grade 1 (n = 155), 375 pg/mL (IQR: 164-981) in grade 2 (n = 858), and 763 pg/mL (IQR: 311-1,915) in grade 3 (n = 474). The y-axis is displayed on a logarithmic scale to accommodate the wide range of NT-proBNP values. AI-ECG = artificial intelligence–enabled electrocardiogram; NT-proBNP = N-terminal pro–B-type natriuretic peptide.

NT-proBNP levels rose step-wise with AI-ECG grade (Figure 1): median 129 pg/mL (IQR: 60-304 pg/mL; n = 2,112) in grade 0, 216 pg/mL (IQR: 87-520 pg/mL; n = 155) in grade 1, 375 pg/mL (IQR: 164-981 pg/mL; n = 858) in grade 2, and 763 pg/mL (IQR: 311-1,915 pg/mL; n = 474) in grade 3 (*P* < 0.001) (Figure 1).

### Echocardiographic indices

In total, 6,232 echocardiographic studies were analyzed. Across increasing AI-ECG diastolic grades, markers of left and right heart function demonstrated progressively unfavorable profiles (Table 2). LA conduit strain, right ventricular (RV) free-wall longitudinal strain (LS), and tricuspid annular plane systolic excursion each showed stepwise reductions. Median LA conduit strain declined from 21.2% (IQR: 15.1-27.3) in grade 0 to 7.1% (IQR: 1.0-14.4) in grade 3 (*P* < 0.001); RV free-wall LS fell from 23.9% (18-27.5) to 14% (6.4-19.4) (*P* < 0.001); and tricuspid annular plane systolic excursion decreased from 20 mm (16-26) to 13 mm (0-17) (*P* < 0.001). In contrast, E, lateral E/e′ ratio, and TR velocity rose across grades (all *P* < 0.001). Spearman analysis showed the strongest inverse correlations between the continuous AI-ECG filling-pressure score and LA conduit strain (ρ = −0.52) and RV free-wall LS (ρ = −0.50). Positive, more modest correlations were observed for mitral A velocity (ρ = 0.35) and lateral E/e′ (ρ = 0.33).

**Table 2:** Echocardiographic Measurements by AI-ECG Diastolic Grade and Correlation With AI-ECG Estimated Filling Pressures

	N	AI-ECG Diastolic Grade 0	AI-ECG Diastolic Grade 1	AI-ECG Diastolic Grade 2	AI-ECG Diastolic Grade 3	*P* Value	Correlation AI-ECG Filling Pressure
LA conduit strain (%)	1,719	21.2 (15.1-27.3)	15.8 (7.7-20.7)	9.7 (0.9-16.9)	7.1 (1-14.4)	<0.001	−0.52 (−0.55 to −0.49)
RV free-wall longitudinal strain (%)	2,262	23.9 (18-27.5)	21.4 (12.5-26.7)	14.7 (2.3-23.1)	14 (6.4-19.4)	<0.001	−0.50 (−0.53 to −0.47)
TAPSE (mm)	3,273	20 (16-26)	19 (13-24)	16 (5-20)	13 (0-17)	<0.001	−0.43 (−0.46 to −0.41)
RA conduit strain (%)	1870	17.8 (11.6-25.7)	16.1 (7.2-23.9)	12.2 (6.5-20.2)	9.3 (4.4-13.9)	<0.001	−0.41 (−0.44 to −0.37)
LV LAX strain (%)	1,544	20.3 (18.8-22.4)	20.3 (18.8-22.4)	18 (14.6-20.1)	15.9 (11.8-19.3)	<0.001	−0.39 (−0.43 to −0.35)
LV 2-chamber strain (%)	1,108	20.4 (18.7-22.6)	20.1 (17.8-22)	18.5 (15.5-21.2)	15.8 (12.7-19.7)	<0.001	−0.36 (−0.40 to −0.31)
RV FAC (%)	2,596	28 (0.4-41.3)	27 (0.38-38)	15 (0-31)	16 (0.13-27.9)	<0.001	−0.35 (−0.39 to −0.32)
Mitral A velocity (m/s)	5,201	0.50 (0.4-0.7)	0.80 (0.6-1)	0.80 (0.5-1.4)	0.7 (0.5-12)	<0.001	0.35 (0.32-0.37)
RA reservoir strain (%)	2061	31.3 (22-41.4)	31.9 (22.6-41.3)	26.7 (16.9-38)	18.1 (11.5-30.4)	<0.001	−0.34 (−0.38 to −0.30)
IVC inspiration diameter (mm)	2,202	9 (7-12)	8 (6-12)	11 (8-17)	14 (9-19)	<0.001	0.33 (0.29-0.36)
Lateral E/e′ ratio	4,029	6.3 (5-8.2)	7.1 (5.5-9.3)	8.1 (6-11.5)	9.1 (6.4-13.7)	<0.001	0.33 (0.30-0.35)
LV ejection fraction (2D or M-mode) (%)	5,831	61 (57-65)	61 (56-66)	58 (50-63)	56 (45-62)	<0.001	−0.24 (−0.26 to −0.21)
LV end-systolic volume index (mL/m^2^)	1854	20 (16-25)	19 (17-22.6)	23.6 (19-31)	28 (19.8-37.9)	<0.001	0.23 (0.19-0.27)
LA volume index (mL/m^2^)	5,324	26 (20-33)	26 (20-35.8)	32 (15-47)	42 (21-65)	<0.001	0.22 (0.19-0.24)
Medial e′ velocity (m/s)	4,578	0.10 (0.08-0.12)	0.08 (0.05-0.11)	0.09 (0.05-1.27)	0.08 (0.05-0.85)	<0.001	−0.20 (−0.22 to −0.17)
Medial E/e′ ratio	4,899	8.6 (6.7-11.1)	10 (6.4-12)	10 (1-15.7)	12 (1-18.3)	<0.001	0.18 (0.15-0.20)
LV end-systolic diameter (mm)	5,505	30 (27-33)	30 (25-34)	33 (28-45)	35 (29-48.5)	<0.001	0.21 (0.18-0.23)
RV end-systolic area (cm^2^)	2,123	19.54 (12.9-27.4)	17.68 (8.6-28)	7.63 (0-20.2)	15.7 (1-28.1)	<0.001	−0.21 (−0.25 to −0.17)
Mitral E velocity (m/s)	5,679	0.80 (0.70-1)	0.75 (0.60-1.02)	0.90 (0.70-1.30)	1.10 (0.80-1.72)	<0.001	0.18 (0.16-0.21)
Tricuspid regurgitation velocity (m/s)	5,371	2.5 (2.3-2.8)	2.5 (2.21-2.8)	2.7 (2.2-3.2)	2.9 (2.2-3.5)	<0.001	0.17 (0.15-0.20)
RV basal diameter (mm)	2,257	45 (37-55)	41.50 (31-54.8)	38 (2-49)	41 (2-53)	<0.001	−0.17 (−0.21 to −0.14)
Mitral deceleration time (ms)	5,207	180 (160-209)	194 (155-230)	170 (110-210)	148 (61-182)	<0.001	−0.17 (−0.20 to −0.15)
RV global longitudinal strain (%)	2,219	21.7 (18.2-25.3)	22.3 (17.2-27.1)	20.5 (9-27.3)	18.6 (11-30.4)	<0.001	−0.14 (−0.18 to −0.10)
RA volume index (mL/m^2^)	2,236	34 (22-50)	31.50 (19-50)	32 (19-50)	42 (27-63)	<0.001	0.09 (0.05-0.13)
LV end-diastolic volume index (mL/m^2^)	1,868	50 (41-61)	44 (36.5-55.5)	44.8 (35.1-58)	51 (39-67)	<0.001	−0.08 (−0.13 to −0.04)

Values are median (IQR) unless otherwise indicated. Echocardiographic variables are shown by AI-ECG diastolic grade (0-3), with values reported as median (IQR). *P* values reflect group differences via Kruskal-Wallis tests. The final column shows Spearman correlation coefficients between each variable and the AI-ECG–estimated filling pressure. Higher diastolic grades were associated with worsening chamber strain, elevated filling pressures, and impaired ventricular function.

E = early diastolic mitral (or left atrioventricular) valve inflow velocity; e′ = early diastolic mitral (or left atrioventricular) annulus velocity; FAC = fractional area change; IVC = inferior vena cava; LA = left atrial; LAX = long-axis; LV = left ventricular; RA = right atrial; RV = right ventricular; TAPSE = tricuspid annular plane systolic excursion; other abbreviation as in Table 1.

### Cardiac catheterization

Among the 2,459 patients who underwent cardiac catheterization during the year of their index ECG, invasive hemodynamics worsened in step with higher AI-ECG diastolic grades (Table 3). Right-atrial/superior vena cava pressure rose from a median 8 mm Hg (IQR: 6-11) in grade 0 to 13 mm Hg (9.5-18) in grade 3 (*P* < 0.001), and subpulmonic ventricular end-diastolic pressure from 10 mm Hg (8-13) to 14 mm Hg (10-19) (*P* < 0.001). Pulmonary pressures increased in parallel, notably PA systolic 30 mm Hg (24-40) to 47 mm Hg (34-63.8), PA diastolic 11 mm Hg (8-14) to 18 mm Hg (13.3-25.8), and PA mean 19 mm Hg (15-24) to 27 mm Hg (19-38) (all *P* < 0.001). PAWP climbed from 11 mm Hg (8-14) in grade 0 to 16 mm Hg (12-21) in grade 3 (*P* < 0.001). By contrast, the rise in subaortic ventricular end-diastolic pressure was 13 mm Hg (10-17) to 14 mm Hg (10.8-18) (*P* = 0.042). Systemic hemodynamics trended downward: mean arterial pressure declined from 87 mm Hg (76-96) to 83 mm Hg (74-91) (*P* < 0.001) and systolic pressure from 115 mm Hg (98-129) to 112 mm Hg (98-126) (*P* = 0.029). Oxygen saturations showed a consistent fall; for example, mixed-venous saturation decreased from 72% (67-77) in grade 0 to 65% (58.6-71) in grade 3 (*P* < 0.001). Flow indices followed the same pattern. The systemic flow index fell from 2.5 L min^−1^ m^−2^ (2.1-3.0) to 2.2 L min^−1^ m^−2^ (1.8-2.8) (*P* < 0.001), and the PVR index increased from 2.6 mmHg L^−1^ min m^−2^ (1.6-4.0) to 3.7 mmHg L^−1^ min m^−2^ (1.9-6.8) (*P* < 0.001). In contrast, the systemic vascular resistance index remained unchanged across grades (*P* = 0.881).

**Table 3:** Cardiac Catheterization Measurements by AI-ECG Diastolic Grade

	N	AI-ECG Diastolic Grade 0	AI-ECG Diastolic Grade 1	AI-ECG Diastolic Grade 2	AI-ECG Diastolic Grade 3	*P* Value
RA/SVC pressure (mm Hg)	1,714	8 (6-11)	8 (5-11)	11 (8-15)	13 (9.5-18)	<0.001
RV systolic pressure (mm Hg)	1,687	39 (30-58)	35 (28-51)	59 (40-84)	60 (46-80)	<0.001
RV end-diastolic pressure (mm Hg)	1,690	10 (8-13)	10 (7-13.3)	12 (9-17)	14 (10-19)	<0.001
PA systolic pressure (mm Hg)	1,664	30 (24-40)	30.5 (25-41)	40 (29-57)	47 (34-64)	<0.001
PA diastolic pressure (mm Hg)	1,660	11 (8-14)	11 (8-14.8)	15 (11-24)	18 (13-26)	<0.001
PA mean pressure (mm Hg)	1,814	19 (15-24)	18 (15-25)	23 (17-34)	27 (19-38)	<0.001
Pulmonary artery wedge pressure (mm Hg)	1,458	11 (8-14)	10 (7-13)	12.5 (10-17)	16 (12-21)	<0.001
LV end-diastolic pressure (mm Hg)	1,368	13 (10-17)	13 (10-17)	14 (10-18)	14 (10.8-18)	0.042
Systolic blood pressure (mm Hg)	1,638	115 (98-129)	120 (108-134)	116 (100-131)	112 (98-126)	0.029
Diastolic blood pressure (mm Hg)	1,631	67 (58-75)	66 (61-77)	66 (58-74)	63 (56-71)	<0.001
Mean blood pressure (mm Hg)	1,622	87 (76-96)	88 (80-99)	86 (76-95)	83 (74-91)	<0.001
Mixed venous saturation (%)	1,189	72 (67-77)	71 (64-75)	68 (61-73)	65 (58.6-71)	<0.001
Pulmonary artery saturation (%)	1,649	75 (69-80)	71 (66-75.8)	70 (64-74.8)	68 (61-74)	<0.001
Pulmonary vein saturation (%)	641	97 (95-99)	97 (95-99)	96 (94-97.8)	96 (91.8-98)	<0.001
Aortic saturation (%)	1,454	97 (94-99)	96 (94-98)	94 (90-97)	94 (90-96)	<0.001
Qs (L/min/m^2^)	1,167	2.5 (2.1-3.0)	2.3 (2.0-3.0)	2.4 (1.9-2.8)	2.2 (1.8-2.8)	<0.001
PVR index (mm Hg/[L/min/m^2^])	941	2.6 (1.6-4.0)	3.4 (2.2-5.1)	3.1 (2.0-5.9)	3.7 (1.9-6.8)	<0.001
SVR index (mm Hg/[L/min/m^2^])	551	32 (24-42)	36 (24-40)	32 (25-41)	31 (24-42)	0.881

This table presents a comprehensive comparison of invasive hemodynamic and oxygen saturation parameters stratified by AI-ECG diastolic grade. For each variable, the total number of observations is reported, followed by the number of patients and the median (IQR) for grades 0, 1, 2, and 3. *P* values from the Kruskal-Wallis test indicate statistically significant differences across the diastolic grades.

PA = pulmonary artery; PVR = pulmonary vascular resistance; Qs = systemic flow index; SVC = superior vena cava; SVR = systemic vascular resistance; other abbreviations as in Tables 1 and 2.

### Correlation of AI-ECG continuous diastolic score with hemodynamics

In the overall cohort, the continuous AI-ECG score correlated strongly with right-sided pressures, RV systolic pressure (ρ = 0.43, *n* = 1,688) and RA pressure (ρ = 0.43, *n* = 1,715), and with PA diastolic pressure (ρ = 0.41, *n* = 1,661). PAWP showed a moderate association (ρ = 0.33, *n* = 1,460); LV end-diastolic pressure was weakly related (ρ = 0.09, *n* = 1,124). Subgroup-specific coefficients and sample sizes appear in Supplemental Table 3.

### Survival analysis

**Figure 2:**
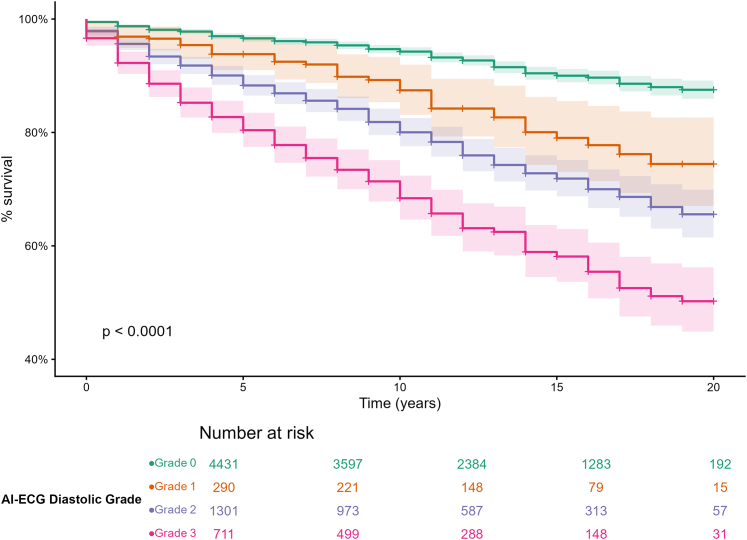
**Kaplan-Meier Survival Curves for Entire Cohort Comparing Survival Probabilities Across Artificial Intelligence–Enabled Electrocardiogram Estimated Diastolic Grades (0-3)** The log-rank test shows a statistically significant difference in survival (*P* < 0.0001). Abbreviation as in Figure 1.

Kaplan-Meier curves (Figure 2) revealed significant survival separation across AI-ECG grades (log-rank *P* < 0.0001). Equivalent curves for the principal CHD subgroups are presented in Supplemental Figures 1 to 7. Patients with right-heart/conotruncal, left-heart, other CHD, and shunt lesions all exhibited highly significant differences (each log-rank *P* < 0.0001). Impressively, patients with systemic right ventricles (log-rank *P* = 0.0025) and post-Fontan palliation (log-rank *P* = 0.026), showed significant separation. Survival did not differ in unrepaired cyanotic disease (log-rank *P* = 0.12), although grade 0 vs grades 2 to 3 curves diverged visually in the unrepaired cyanotic disease cohort.

### Association between AI-ECG diastolic grade and survival: Cox regression analysis

Table 4 summarizes Cox regression results. Univariate analysis showed progressively higher hazard with worsening grade (grade 1 HR: 2.39; 95% CI: 1.53-3.55; grade 2 HR: 2.68; 95% CI: 2.16-3.33; grade 3 HR: 4.75; 95% CI: 3.8-5.93; all *P* < 0.001). As shown in Table 4, the majority of variables were statistically significant, for example, the univariate predictors age (HR: 1.04 per year), male sex (HR: 1.28 [1.07-1.53]), heart failure (HR: 2.61 [2.14-3.19]), chronic kidney disease (HR: 3.97 [3.13-5.05]), cirrhosis (HR: 4.47 [3.21-6.22]), and log-transformed NT-proBNP (HR: 1.58 [1.45-1.72]) (all *P* < 0.001).

**Table 4:** Univariate and Multivariate Cox Regression Analysis

	Level	Univariate			Multivariate		
		HR	95% CI	*P* Value	HR	95% CI	*P* Value
AI- ECG diastolic grade (reference: Grade 0)	AI-ECG diastolic grade 1	2.39	1.53-3.55	<0.001	1.33	0.86-2.06	0.20
	AI-ECG diastolic grade 2	2.68	2.16-3.33	<0.001	1.38	1.09-1.75	0.007
	AI-ECG diastolic grade 3	4.75	3.8-5.93	<0.001	1.63	1.27-2.08	<0.001
Age at first visit (y)	-	1.04	1.04-1.05	<0.001	1.03	1.03-1.04	<0.001
log(NT-proBNP)	-	1.58	1.45-1.72	<0.001	1.54	1.45-1.64	<0.001
Sex (reference: female)	Male	1.28	1.07-1.53	0.006	1.41	1.17-1.69	<0.001
Congenital heart disease Group (reference: RH/conotruncal)	Left heart disease	0.95	0.72-1.26	0.733	0.95	0.69-1.30	0.74
	Shunt lesion	1.55	1.22-1.97	<0.001	1.11	0.83-1.49	0.48
	Cyanotic heart disease	4.58	3.27-6.41	<0.001	2.96	1.38-6.34	0.005
	Other CHD	2.17	1.06-4.44	0.034	1.35	0.63-2.90	0.44
	Systemic right ventricle	1.11	0.77-1.6	0.577	0.69	0.32-1.50	0.34
	Fontan palliation	2.23	1.62-3.06	<0.001	2.81	1.28-6.17	0.01
Congenital heart disease severity (reference: mild)	Moderate	0.84	0.67-1.05	0.132	1.26	0.94-1.68	0.12
	Complex	1.39	1.08-1.78	0.011	1.39	0.64-3.05	0.41
Heart failure	-	2.61	2.14-3.19	<0.001	1.33	1.07-1.66	0.01
Atrial arrhythmia	-	2.25	1.89-2.69	<0.001	1.23	0.88-1.73	0.22
Atrial fibrillation	-	2.77	2.31-3.32	<0.001	1.22	0.89-1.68	0.21
Atrial flutter/tachycardia	-	1.48	1.19-1.84	<0.001	0.81	0.62-1.07	0.14
Ventricular arrhythmia	-	1.60	1.2-2.12	<0.001	0.82	0.51-1.31	0.40
Sustained ventricular tachycardia	-	2.06	1.33-3.19	<0.001	1.48	0.86-2.55	0.15
Nonsustained ventricular tachycardia	-	1.25	0.86-1.81	0.24	0.88	0.52-1.49	0.64
Ventricular fibrillation/cardiac arrest	-	2.66	1.59-4.45	<0.001	1.49	0.82-2.73	0.19
Hypertension	-	1.62	1.35-1.95	<0.001	0.84	0.68-1.05	0.12
Diabetes	-	2.13	1.68-2.7	<0.001	1.33	1.01-1.74	0.039
Smoking history	-	1.70	1.39-2.08	<0.001	1.20	0.97-1.49	0.10
Obesity	-	0.99	0.8-1.23	0.942	1.01	0.80-1.27	0.94
Coronary artery disease	-	1.85	1.39-2.48	<0.001	1.08	0.79-1.47	0.64
Venous thrombosis	-	2.15	1.38-3.37	<0.001	1.11	0.69-1.79	0.67
Left heart thrombus	-	3.79	2.03-7.09	<0.001	1.78	0.90-3.52	0.10
Stroke	-	2.00	1.47-2.71	<0.001	1.28	0.93-1.76	0.14
Pulmonary embolism	-	2.18	1.12-4.21	0.021	2.35	1.17-4.69	0.016
Obstructive sleep apnea	-	1.48	1.19-1.83	<0.001	0.93	0.72-1.19	0.55
Chronic kidney disease	-	3.97	3.13-5.05	<0.001	1.09	0.82-1.45	0.57
Infective endocarditis	-	1.66	1.11-2.49	0.013	0.85	0.56-1.30	0.46
Protein losing enteropathy	-	2.49	1.18-5.25	0.017	1.28	0.58-2.87	0.54
Cirrhosis	-	4.47	3.21-6.22	<0.001	2.59	1.73-3.89	<0.001

This table summarizes the univariate and multivariate Cox regression analysis. HRs with 95% CIs and *P* values are reported.

NT-proBNP = N-terminal pro–B-type natriuretic peptide; RH = right heart; other abbreviations as in Table 1.

**Figure 3:**
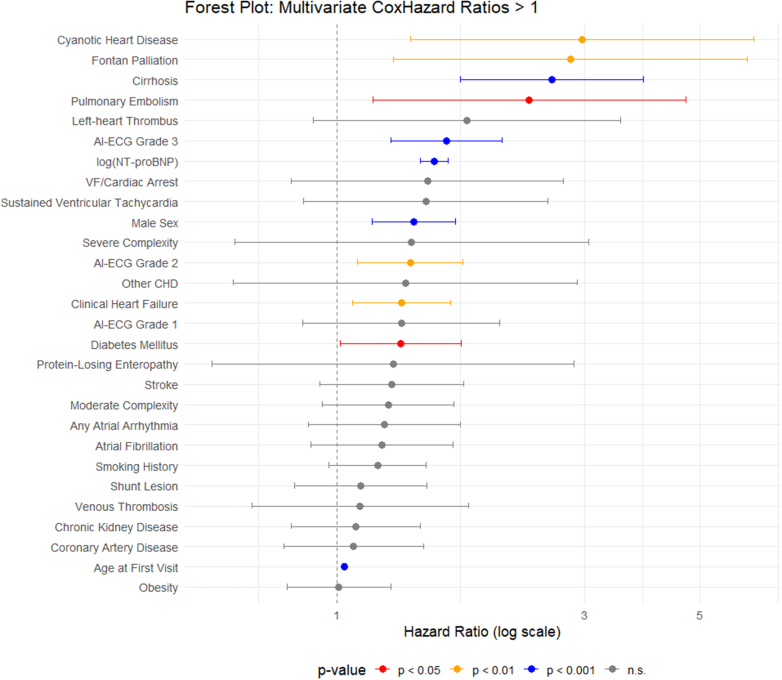
**Forest Plot of Multivariate Cox Regression Analysis for Predictors of Mortality** This plot summarizes HRs (HR > 1) from the multivariate Cox regression model, illustrating independent risk factors associated with mortality in the study cohort. Point estimates represent adjusted HRs, with 95% CIs depicted as horizontal lines. CHD = congenital heart disease; VF = ventricular fibrillation; other abbreviations as in Figure 1.

Multivariate analysis confirmed AI-ECG grade as an independent predictor of mortality: HR 1.38 (1.09-1.75; *P* = 0.007) for grade 2 and HR 1.63 (1.27-2.08; *P* < 0.001) for grade 3 vs grade 0, whereas grade 1 was not significant (HR: 1.33 [0.86-2.06]; *P* = 0.20). Each additional year of age conferred a 3% higher risk (HR: 1.03; *P* < 0.001), and male sex remained adverse (HR: 1.41 [1.17-1.69]; *P* < 0.001). Log-NT-proBNP retained a strong association (HR: 1.54 [1.45-1.64]; *P* < 0.001). Relative to right heart/conotruncal lesions, cyanotic CHD (HR: 2.96 [1.38-6.34]; *P* = 0.005) and Fontan palliation (HR: 2.81 [1.28-6.17]; *P* = 0.010) carried excess risk. Heart failure (HR: 1.33 [1.07-1.66]; *P* = 0.010), diabetes (HR: 1.33 [1.01-1.74]; *P* = 0.039), pulmonary embolism (HR: 2.35 [1.17-4.69]; *P* = 0.016), and cirrhosis (HR: 2.59 [1.73-3.89]; *P* < 0.001) were also independently predictive (Figure 3). The final model showed excellent discrimination (concordance = 0.83 [95% CI: 0.81-0.85] with negligible multicollinearity.

### AI-ECG filling pressure model’s performance and calibration at predicting elevated PAWP

**Figure 4:**
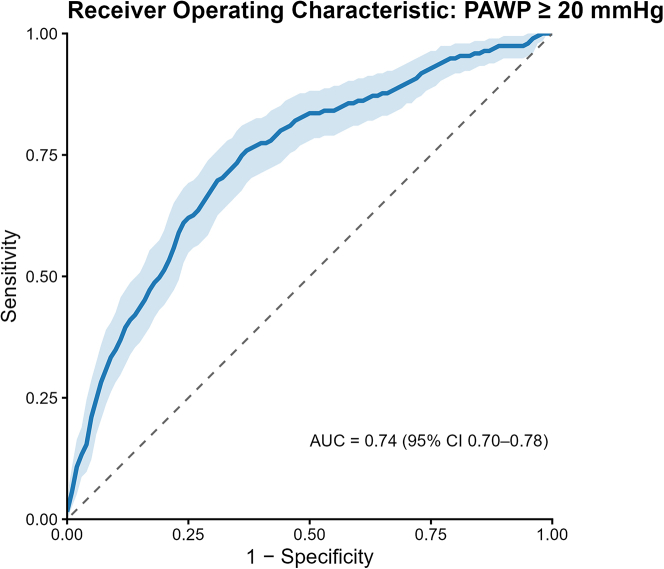
**Receiver Operating Characteristic Curve of the Artificial Intelligence–Enabled Electrocardiogram Model for Detecting Elevated Pulmonary Artery Wedge Pressure ≥20 mmHg** The model achieved an area under the receiver operating characteristic curve (AUC) of 0.74 (95% CI: 0.70-0.78). PAWP = pulmonary artery wedge pressure.

Diagnostic performance of AI-ECG–predicted PAWP at candidate hemodynamic cutoffs (15, 18, 20, 22 mm Hg) are reported in Supplemental Table 4. The AI-ECG probability yielded an AUC of 0.74 (95% CI: 0.70-0.78) for detecting PAWP ≥20 mm Hg (Figure 4). Before recalibration, the model’s probabilities were systematically too high (Brier score = 0.27, calibration intercept = −1.95, calibration slope = 0.29). After recalibration, the calibration intercept approached zero and the slope approached 1, indicating excellent agreement between predicted and observed risk. A locally estimated scatterplot smoothing–smoothed calibration plot was generated for the full cohort postrecalibration and demonstrates good agreement in the mid-range of predicted risk, with modest overestimation at higher predicted probabilities (Supplemental Figure 8). Subgroup analysis showed marked heterogeneity in discrimination, with AUCs ranging from 0.54 in patients with systemic right ventricles to 0.86 in patients with left heart disease, and similar patterns in calibration (Table 5). All groups exhibited overprediction and overdispersion before recalibration; the single recalibration step corrected both issues across groups. These findings suggest that although the model’s discrimination was moderate to strong in some subgroups, recalibration helped substantially reduce miscalibration and improve probabilistic accuracy in all anatomical groups.

**Table 5:** Discrimination and Calibration of the Original and Recalibrated AI-ECG Model for Elevated PAWP ≥20 mm Hg by CHD Subgroup

	AUC (95% CI)	Calibration Intercept (95% CI)	Calibration Slope (95% CI)	Brier (Original) (95% CI)	Brier^a^ (Recalibrated) (95% CI)
Overall (N = 1,460)	0.74 (95% CI: 0.70-0.78)	−1.95 (95% CI: −2.12 to −1.78)	0.29 (95% CI: 0.23-0.35)	0.27 (95% CI: 0.26-0.29)	0.11 (95% CI: 0.09-0.12)
RH/conotruncal defects (n = 464)	0.70 (95% CI: 0.60-0.80)	−2.60 (95% CI: −2.99 to −2.21)	0.26 (95% CI: 0.11-0.41)	0.21 (95% CI: 0.19-0.24)	0.06 (95% CI: 0.04-0.07)
Left heart disease (n = 177)	0.86 (95% CI: 0.80-0.92)	−1.07 (95% CI: −1.50 to −0.63)	0.46 (95% CI: 0.31-0.62)	0.19 (95% CI: 0.14-0.23)	0.14 (95% CI: 0.11-0.17)
Shunt lesion (n = 441)	0.74 (95% CI: 0.67-0.81)	−1.62 (95% CI: −1.90 to −1.34)	0.28 (95% CI: 0.18-0.37)	0.21 (95% CI: 0.18-0.24)	0.11 (95% CI: 0.09-0.13)
Cyanotic heart disease (n = 56)	0.69 (95% CI: 0.53-0.85)	−1.72 (95% CI: −2.60 to −0.84)	0.31 (95% CI: 0.00-0.63)	0.38 (95% CI: 0.29-0.48)	0.16 (95% CI: 0.10-0.22)
Other CHD^b^ (n = 5)		0.00^b^	0.00^b^	0.02 (95% CI: 0.00-0.07)	0.11 (95% CI: 0.00-0.31)
Systemic right ventricle (n = 103)	0.54 (95% CI: 0.42-0.67)	−0.91 (95% CI: −1.45 to −0.38)	0.07 (95% CI: −0.12 to 0.26)	0.46 (95% CI: 0.39-0.53)	0.21 (95% CI: 0.18-0.25)
Fontan palliation (n = 214)	0.64 (95% CI: 0.39-0.88)	−3.63 (95% CI: −4.60 to −2.66)	0.17 (95% CI: −0.15 to 0.50)	0.49 (95% CI: 0.44-0.54)	0.03 (95% CI: 0.01-0.06)

AUC = area under the receiver operating characteristic curve; PAWP = pulmonary artery wedge pressure; other abbreviations as in Tables 1 and 4.

a Brier score after logistic recalibration.

b Very small subgroup (n = 5; 1 event). AUC CI not estimable; intercept/slope omitted to avoid unstable estimates.

## Discussion

This study extends a validated AI-ECG algorithm for diastolic dysfunction to the largest ACHD cohort studied to date. AI-ECG correlated with clinical, echo, and invasive measures of elevated cardiac filling pressures. We show that the AI-derived diastolic grade stratifies clinical risk and complexity, mirrors invasive hemodynamic burden, and independently predicts mortality across a broad anatomic spectrum. Several findings, however, diverge from prior work in noncongenital populations and underscore how congenital anatomy reshapes the pathophysiology that the algorithm detects.

The original neural network was trained on 4 echocardiographic features; e′, E/e′, LA volume index, and TR velocity. TR velocity is the Doppler surrogate for RV systolic pressure and was included to screen for pulmonary hypertension in structurally normal hearts. In ACHD, right-sided disease and surgically altered RV geometry are much more prevalent than isolated LV diastolic dysfunction, so the model’s weighting of TR velocity amplifies its sensitivity to RA, RV, and PA pressures. Subgroup analysis supports this interpretation. For example, in lesions in which left-heart loading drives hemodynamics, namely left-heart disease and large shunts, correlations with PAWP were strongest (ρ ≈ 0.56 and ρ ≈ 0.45) (Supplemental Table 3). Conversely, correlations were attenuated in cyanotic hearts and Fontan circulations, where pulmonary vascular physiology and chronic hypoxemia obscure conventional diastolic markers.

In relation to assessing the correlation of echocardiographic parameters of dysfunction in our patient cohort, we acknowledge that although the ratio of E/e′ is clinically useful as a measure of filling pressures, E/e′ has limitations that limits its accuracy as a standalone measure. Foremost is the assumption that mitral annulus velocities represent global ventricular relaxation. This is not true for patients with segmental dysfunction or for patients with ACHD among whom scars and patches from surgical repair and valve replacement are common. E/e′ is also influenced by atrial and ventricular volumes, pressure, and relaxation. Rather than relying on E/e′ as a standalone measure of diastolic function, current guidelines suggest several parameters that can be used to generate an overall grading of diastolic function. This reinforces the need for multiparameter or machine-learning approaches rather than single metrics such as E/e′ in ACHD. Automated speckle-tracking measures, including LA strain and RV free-wall strain, which showed the strongest inverse associations may deserve greater weight in congenital laboratories.

Kaplan-Meier survival analysis further substantiated the clinical relevance of the AI-ECG diastolic dysfunction model. Significant differences in survival were noted across the 4 AI-ECG diastolic grades in the overall cohort (Central Illustration). Notably, subgroup analyses revealed that these differences were highly significant in patients with right heart/conotruncal lesions, left heart disease, shunt lesions, other CHD, a systemic right ventricle, and those post-Fontan palliation, whereas the survival stratification was less distinct in patients with cyanotic heart disease. These findings suggest that although the model performs robustly in many ACHD subgroups, its predictive accuracy may be reduced in populations with ECG patterns that differ substantially from those in the original training data and additionally may reflect complex or atypical hemodynamic profiles.

**Central Illustration:**
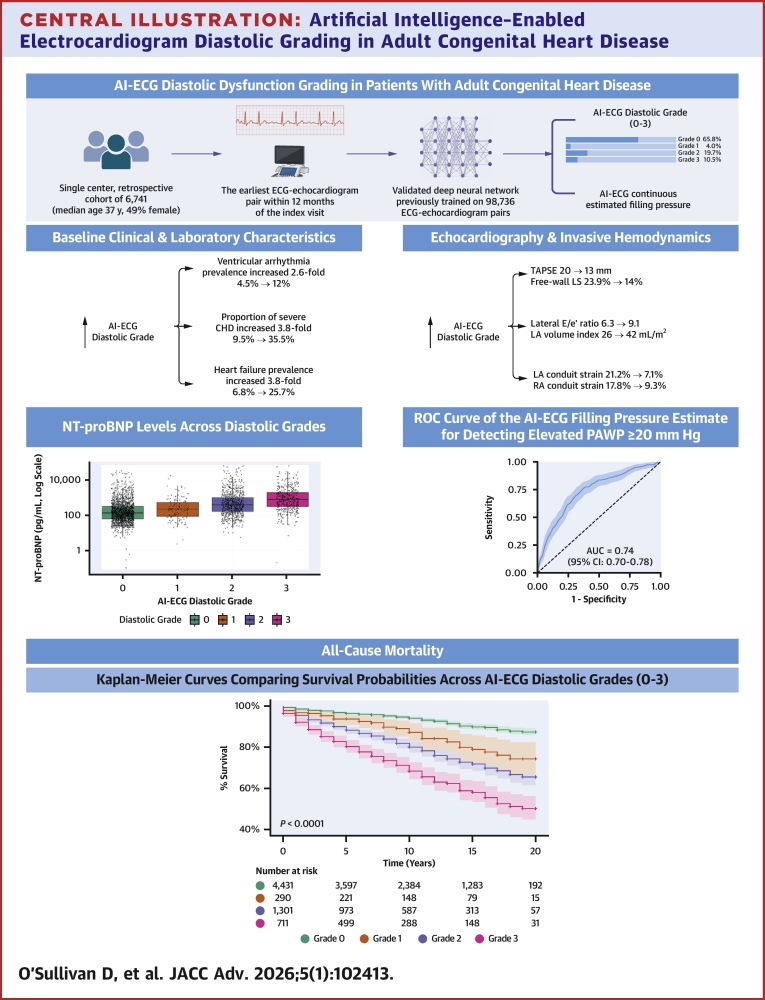
**Artificial Intelligence–Enabled Electrocardiogram Diastolic Grading in Adult Congenital Heart Disease** Single-center retrospective cohort of 6,741 adults with CHD using the earliest ECG-echocardiogram pair (≤12 months); a validated deep neural network produced an AI-ECG diastolic grade (0-3) and a continuous filling-pressure estimate. Higher grades were associated with greater disease burden, stepwise increase in NT-proBNP, worse echocardiographic/hemodynamic indices, and independently associated with mortality in multivariate analysis and furthermore a stepwise lower survival across grades (log-rank *P* < 0.001). AI-ECG = artificial intelligence–enabled electrocardiogram; E = early diastolic mitral (or left atrioventricular) valve inflow velocity; e′ = early diastolic mitral (or left atrioventricular) annulus velocity; ECG = electrocardiogram; LA = left atrial; LS = longitudinal strain; NT-proBNP = N-terminal pro–B-type natriuretic peptide; PAWP = pulmonary artery wedge pressure; RA = right atrial; ROC = receiver operating characteristic curve; TAPSE = tricuspid annular plane systolic excursion.

AI-ECG diastolic grades were associated with clinical markers of heart failure and independently predicted survival. Our prior work has shown that AI-ECG is influenced by both cardiac and noncardiac conditions, including cirrhosis and chronic kidney disease.^22^^,^^23^ These findings suggest that the current AI-ECG model for diastolic dysfunction may stratify mortality risk by capturing contributions from both elevated cardiac filling pressures and extracardiac comorbidities. Consistent with this, established predictors of all-cause mortality in ACHD cohorts, such as heart failure, male sex, and older age, emerged as independent risk factors in our analysis.^1^, ^2^, ^3^ Cirrhosis and pulmonary embolism also conferred an increased mortality risk, underscoring the contribution of hepatic congestion and thrombotic complications in advanced ACHD.^18^^,^^24^ In addition, diabetes mellitus was associated with a 41% increase in mortality, aligning with its growing recognition as a risk modifier in congenital populations. Log-transformed NT-proBNP remained a strong independent predictor, reinforcing its prognostic utility across both acquired and congenital cardiovascular disease settings.^25^

Standard echocardiographic algorithms for diastolic grading assume a normal LV configuration and load conditions; when applied to systemic RVs, single ventricles, or patch-repaired chambers, those assumptions break down and yield conflicting or uninterpretable results. Our original AI-ECG model was trained to reproduce the echo-derived diastolic-dysfunction grade in patients with conventional cardiac anatomy. Validating it against an invasive measure of filling pressure therefore provides a more rigorous test. In the original development cohort, the AI-ECG demonstrated excellent discrimination for echo-derived increased filling pressure (AUC: 0.911; 95% CI: 0.909-0.914), with a sensitivity of 83.2%, specificity of 82.9%, positive predictive value of 58%, and negative predictive value of 94.5% with a prevalence of 22.2%. When applied to an invasive hemodynamic endpoint (PAWP ≥20 mm Hg), discrimination was lower but still performed well (AUC: 0.75; 95% CI: 0.70-0.80) (Figure 4). This attenuation likely reflects the stricter reference standard, decreased prevalence, and the broader spectrum of loading conditions in ACHD. After a single recalibration step, there was acceptable calibration across diverse anatomic subgroups. Most importantly, the AI-ECG–estimated diastolic-dysfunction grade itself remained an independent predictor of mortality, underscoring that the network is capturing prognostically meaningful electrical signatures. Importantly this method allows for grading of diastolic dysfunction in those who have indeterminate grading on echocardiogram. This finding positions AI-ECG as the potential frontline tool for identifying patients with ACHD with adverse ventricular loading who warrant closer surveillance or invasive assessment.

### Study Limitations

Despite these promising results, several limitations warrant consideration. The retrospective single-center design may limit generalizability, and the tertiary care setting of our ACHD population might introduce referral bias. In addition, the temporal gap between the AI-ECG and corresponding echocardiographic or invasive hemodynamic assessments, occasionally extending up to 1 year, represents a potential limitation, as cardiac physiology can evolve significantly over time. This interval variability may introduce noise into the observed associations and should be addressed in future prospective studies with synchronized data collection. Prospective validation in larger, external cohorts is needed to confirm these findings and to evaluate the model’s utility in predicting ACHD-specific outcomes such as heart failure hospitalizations and mortality.

## Conclusions

The AI-ECG model reliably detects elevated filling pressures in ACHD across a broad anatomic spectrum and remains a strong independent predictor of mortality, even after adjusting for conventional risk factors. Validating the algorithm against invasively measured PAWP demonstrates that the network captures physiologically meaningful electrical signatures of ventricular loading. By revealing subtle ECG waveforms that correlate with invasive hemodynamic burden, the AI-ECG diastolic grade shows promise as a scalable, noninvasive screening tool to guide risk stratification and guideline-directed medical treatment in patients with ACHD, especially those with, and at risk of, heart failure due to elevated filling pressures. Clinical trials are warranted to assess the feasibility of AI-ECG–guided heart failure detection and treatment in patient with ACHD populations receiving care across diverse resource settings.

Perspectives **COMPETENCY IN MEDICAL KNOWLEDGE:** In adults with CHD, noninvasive grading of diastolic dysfunction is difficult due to atypical anatomy and surgical alterations. An AI-enabled 12-lead ECG model provided a 4-tier diastolic grade (0-3) that tracked with biomarkers (NT-proBNP), echocardiographic strain indices, invasive hemodynamics, and independently predicted mortality across diverse ACHD subgroups. Discrimination for PAWP **≥**20 mm Hg was moderate and improved with simple recalibration. These data support AI-ECG as a scalable adjunct to echo and catheterization for risk stratification in ACHD.

**TRANSLATIONAL OUTLOOK:** Prospective, multicenter studies should test AI-ECG–guided pathways (screening, triage to imaging or cath, and longitudinal monitoring) and evaluate clinical utility (heart failure admissions, treatment optimization, and costs). Subgroup-specific calibration and model updates, particularly for systemic RV and Fontan, may further enhance accuracy and equity of deployment in congenital programs.

## Funding support and author disclosures

The Mayo Clinic Adult Congenital Heart Disease Registry is supported by the Al-Bahar Research Grant (10.13039/100000871Mayo Clinic Rochester, Minnesota). Dr Oh serves as a consultant for Medtronic’s valve projects. Drs Friedman, Attia, Lopez-Jimenez, and Oh have invented algorithms licensed to ANUMANA and may benefit from algorithm commercialization via Mayo Clinic. Drs Friedman, Attia, and Lopez-Jimenez are members of the scientific advisory board to ANUMANA. Dr Luke J Burchill is the recipient of a National Health & Medical Research Council of Australia Investigator Award. All other authors have reported that they have no relationships relevant to the contents of this paper to disclose.
