# Supplementary material for: Artificial Intelligence–Enabled ECG for Diastolic Dysfunction in Congenital Heart Disease: A Novel Risk Stratification Tool
Source: JACC Adv. 2025 Dec 16;5(1):102413. doi: 10.1016/j.jacadv.2025.102413 (PMC12869886; doi:10.1016/j.jacadv.2025.102413)
Supplement: Supplementary files [file mmc1.docx]

**Supplementary files**

**Supplementary appendix 1**: The MACHD Registry is a retrospective database of adults with CHD that received care at the Mayo Clinic Enterprise (Mayo Cline Rochester, MN; Mayo Clinic Jacksonville, FL; and Mayo Clinic Scottsdale, AZ). The inclusion criteria were: (1) CHD diagnosis; (2) at least one clinical evaluation in the pediatric or adult CHD clinic in any of the 3 Mayo Clinic sites after age 18 years; (3) at least one echocardiogram in any of the 3 Mayo Clinic sites after age 18 years. In order to avoid an overlap with patients with certain CHD diagnoses that received care in the general cardiology clinic, we excluded patients with the following diagnoses: (1) patients with isolated atrial septal defect repaired prior to the first clinical evaluation in the adult CHD clinic; (2) patients with isolated ventricular septal defect repaired prior to the first clinical evaluation in the adult CHD clinic; (3) patients with isolated patent ductus arteriosus repaired prior to the first clinical evaluation in the adult CHD clinic; (4) patients with isolated bicuspid aortic valve presenting with aortic stenosis after age 18 years. Eligible patients were identified using the Advanced Cohort Explorer, which is an institutionally approved search engine. This software was later replaced by the Mayo Data Explorer (MDE). The electronic heart records were manually reviewed in all cases by data abstractors.

**Supplementary appendix 2A**

**TRIPOD‑AI for Abstracts – Completed Checklist**

| Section / Item | Requirement | How it is addressed in the revised abstract |
| --- | --- | --- |
| Title 1 | Identify the study as developing or evaluating the performance of a multivariable prediction model, the target  population, and the outcome to be predicted | “AI‑Enabled ECG for Diastolic Dysfunction in Congenital Heart Disease: A Non‑Invasive Tool for Risk Stratification” specifies AI model, ACHD population, diastolic dysfunction / filling pressure outcome. |
| Background 2 | Provide a brief explanation of the healthcare context and rationale for developing or evaluating the performance of all models | “Standard echocardiographic criteria… are unreliable in ACHD” (implied in opening sentence of Background). |
| Objectives 3 | Specify the study objectives, including whether the study describes model development, evaluation, or both | “We evaluated whether an AI‑ECG model… predicts survival… and assessed its correlation with echocardiographic and invasive markers.” (performance evaluation). |
| Methods 4 | Describe the sources of data | “Single‑center retrospective study… Mayo Clinic ACHD Registry (2000–2023).” |
| Methods 5 | Describe the eligibility criteria and setting where the data were collected | “6 741 patients… ECG within 12 months of index visit.” (tertiary ACHD centre). |
| Methods 6 | Specify the outcome to be predicted by the model, including time horizon of predictions in case of prognostic models | Primary diagnostic outcome: PAWP > 20 mmHg; prognostic outcome: all‑cause mortality during follow‑up 2000–2023. |
| Methods 7 | Specify the type of model, a summary of the model-building steps, and the method for internal validation | “Validated deep neural network (trained on 98 736 ECG‑echo pairs) assigned an AI‑ECG diastolic grade.” (pre‑existing network; no redevelopment). |
| Methods 8 | Specify the measures used to assess model performance (eg, discrimination, calibration, clinical utility) | “AUC… calibration via logistic recalibration, correlation coefficients.” |
| Results 9 | Report the number of participants and outcome events | “6 741 patients… AI‑ECG graded 65.8 % / 4.0 % / 19.7 % / 10.5 %;” mortality and haemodynamic results reported. |
| Results 10 | Summarise the predictors in the final model | Predictors are ECG waveforms processed by the pre‑trained model; implicitly stated by reference to AI‑ECG probability and grade. |
| Results 11 | Report model performance estimates (with confidence intervals) | AUC 0.75 (95 % CI 0.70–0.80); calibration metrics (intercept, slope) summarised qualitatively. |
| Discussion 12 | Give an overall interpretation of the main results | “AI‑ECG grading correlates with echo & invasive filling pressures and independently predicts mortality… supports its utility as scalable non‑invasive tool.” |
| Registration 13 | Give the registration number and name of the registry or repository | Not applicable (retrospective registry analysis; no preregistration). |

**Supplementary appendix 2B**

**TRIPOD + AI Manuscript Checklist**

| Section / Topic | Item | Checklist item (verbatim) | Reported in manuscript |
| --- | --- | --- | --- |
| TITLE | 1 | Identify the study as developing or evaluating the performance of a multivariable prediction model, the target population, and the outcome to be predicted | Title page p 1 |
| ABSTRACT | 2 | See TRIPOD+AI for Abstracts checklist | Structured abstract p 1 |
| INTRODUCTION | 3a | Explain the healthcare context (including whether diagnostic or prognostic) and rationale for developing or evaluating the prediction model, including references to existing models | Introduction ¶ 1–3  p 3 |
|  | 3b | Describe the target population and the intended purpose of the prediction model in the context of the care pathway, including its intended users (e.g., healthcare professionals, patients, public) | Introduction ¶ 3  p 3 |
|  | 3c | Describe any known health inequalities between sociodemographic groups | Introduction ¶ 2 p 3 |
| Objectives | 4 | Specify the study objectives, including whether the study describes the development or validation of a prediction model (or both) | Intro ¶ 4–5 (“primary aim… secondary aim…”) p 3 |
| METHODS – Data | 5a | Describe the sources of data separately for the development and evaluation datasets (e.g., randomized trial, cohort, routine care or registry data), the rationale for using these data, and representativeness of the data | Methods “Methods” first paragraph + Appendix 1. p 5 |
|  | 5b | Specify the dates of the collected participant data, including start and end of participant accrual; and, if applicable, end of follow-up | Methods first paragraph (“2000–2023”) p 5 |
| Participants | 6a | Specify key elements of the study setting (e.g., primary care, secondary care, general population) including the number and location of centres | Methods first paragraph  p 5 |
|  | 6b | Describe the eligibility criteria for study participants | Methods first paragraph + “AI‑ECG model” subsection p 5 |
|  | 6c | Give details of any treatments received, and how they were handled during model development or evaluation, if relevant | Not applicable (observational) |
| Data preparation | 7 | Describe any data pre‑processing and quality checking, including whether this was similar across relevant sociodemographic groups | Methods “AI‑ECG model” and “Statistical analysis” p 5, p8  (predictor definition) |
| Outcome | 8a | Clearly define the outcome that is being predicted and the time horizon, including how and when assessed, the rationale for choosing this outcome, and whether the method of outcome assessment is consistent across sociodemographic groups | Methods “Mortality” p 7 and “Filling Pressure Model Validation” p8 |
|  | 8b | If outcome assessment requires subjective interpretation, describe the qualifications and demographic characteristics of the outcome assessors | N/A (objective registry & Accurint death data) |
|  | 8c | Report any actions to blind assessment of the outcome | NA |
| Predictors | 9a | Describe the choice of initial predictors (e.g., literature, previous models, all available predictors) and any pre-selection of predictors before model building | Methods “AI‑ECG model” (pre‑trained network) |
|  | 9b | Clearly define all predictors, including how and when they were measured (and any actions to blind assessment of predictors for the outcome and other predictors) | Same subsection; ECG waveform acquisition described p5-6 |
|  | 9c | If predictor measurement requires subjective interpretation, describe the qualifications and demographic characteristics of the predictor assessors | N/A (automated ECG output) |
| Sample size | 10 | Explain how the study size was arrived at (separately for development and evaluation), and justify that the study size was sufficient to answer the research question. Include details of any sample size calculation | Methods first paragraph (registry census), p5 |
| Missing data | 11 | Describe how missing data were handled. Provide reasons for omitting any data | Statistical analysis  p8 and results page 9 |
| Analytical methods | 12a | Describe how the data were used (e.g., for development and evaluation of model performance) in the analysis, including whether the data were partitioned, considering any sample size requirements | Statistical analysis section p8 |
|  | 12b | Depending on the type of model, describe how predictors were handled in the analyses (functional form, rescaling, transformation, or any standardisation). | “AI‑ECG model” (soft‑max aggregation) p5 |
|  | 12c | Specify the type of model, rationale, all model‑building steps, including any hyper‑parameter tuning, and method for internal validation | Cited original dev paper; this study = external validation; internal steps in ref 17 |
|  | 12d | Describe if and how any heterogeneity in estimates of model parameter values and model performance was handled and quantified across clusters (e.g., hospitals, countries). See TRIPOD-Cluster for additional considerations3 | Subgroup calibration by anatomy (Table 5) |
|  | 12e | Specify all measures and plots used (and their rationale) to evaluate model performance (e.g., discrimination, calibration, clinical utility) and, if relevant, to compare multiple models | Statistical analysis (AUC, Brier, LOESS, KM, C‑stat) |
|  | 12f | Describe any model updating (e.g., recalibration) arising from the model evaluation, either overall or for particular sociodemographic groups or settings | “Filling Pressure Model Validation” (logistic recalibration) |
|  | 12g | For model evaluation, describe how the model predictions were calculated (e.g., formula, code, object, application programming interface) | Same subsection (sum of Grade 2 + 3 probabilities), p8-9 |
| Class imbalance | 13 | If class imbalance methods were used, state why and how this was done, and any subsequent methods to recalibrate the model or the model predictions | Not used, recalibrated p8-9 |
| Fairness | 14 | Describe any approaches that were used to address model fairness and their rationale | Discussion last paragraph (limitations & future fairness work) |
| Model output | 15 | Specify the output of the prediction model (e.g., probabilities, classification). Provide details and rationale for any classification and how the thresholds were identified | Filling Pressure Model Validation, Youden index, PAWP. p8-9 |
| Training vs evaluation | 16 | Identify any differences between the development and evaluation data in healthcare setting, eligibility criteria, outcome, and predictors | Introduction p3 |
| Ethical approval | 17 | Name the institutional research board or ethics committee that approved the study and describe the participant-informed consent or the ethics committee waiver of informed consent | Methods first paragraph (IRB #20‑007695; waiver) |
| OPEN SCIENCE | 18a | Give the source of funding and the role of the funders for the present study | “Sources of Funding” p18 |
|  | 18b | Declare any conflicts of interest and financial disclosures for all authors | “Disclosures” p19 |
|  | 18c | Indicate where the study protocol can be accessed or state that a protocol was not prepared | NA |
|  | 18d | Provide registration information for the study, including register name and registration number, or state that the study was not registered | Not registered |
|  | 18e | Provide details of the availability of the study data | Data‑sharing statement in Acknowledgements p19 |
|  | 18f | Provide details of the availability of the analytical code | As above p19 |
| PATIENT & PUBLIC INVOLVEMENT | 19 | Provide details of any patient and public involvement during the design, conduct, reporting, interpretation, or dissemination of the study or state no involvement. | NA |
| RESULTS – Participants | 20a | Describe the flow of participants through the study, including the number of participants with and without the outcome and, if applicable, a summary of the follow-up time. A diagram may be helpful. | Results first paragraph, p9 Flowchart not included. |
|  | 20b | Report the characteristics overall and, where applicable, for each data source or setting, including the key dates, key predictors (including demographics), treatments received, sample size, number of outcome events, follow-up time, and amount of missing data. A table may be helpful. Report any differences across key demographic groups | Table 1, results P 9 |
|  | 20c | For model evaluation, show a comparison with the development data of the distribution of important predictors (demographics, predictors, and outcome). | Discussion p16 |
| Model development | 21 | Specify the number of participants and outcome events in each analysis (e.g., for model development, hyperparameter tuning, model evaluation) | Results p9 |
| Model specification | 22 | Provide details of the full prediction model (e.g., formula, code, object, application programming interface) to allow predictions in new individuals and to enable third-party evaluation and implementation, including any restrictions to access or re-use (e.g., freely available, proprietary) | Proprietary; statement of access restrictions included in acknowledgments |
| Model performance | 23a | Report model performance estimates with confidence intervals, including for any key subgroups (e.g.,sociodemographic). Consider plots to aid presentation | Results (AUC, Brier, CI) + Table 5 |
|  | 23b | If examined, report results of any heterogeneity in model performance across clusters. | Subgroup AUCs in Table 5 |
| Model updating | 24 | Report the results from any model updating, including the updated model and subsequent performance | Results “After recalibration… intercept ≈0, slope ≈1”, p13 |
| DISCUSSION | 25 | Give an overall interpretation of the main results, including issues of fairness in the context of the objectives and previous studies | Discussion p14 |
| Limitations | 26 | Discuss any limitations of the study (such as a non-representative sample, sample size, overfitting, missing data) and their effects on any biases, statistical uncertainty, and generalizability | Discussion p17 |
| Usability in care | 27a | Describe how poor quality or unavailable input data (e.g., predictor values) should be assessed and handled when implementing the prediction model | NA, p 17 applicable for indeterminate echocardiographic |
|  | 27b | Specify whether users will be required to interact in the handling of the input data or use of the model, and what level of expertise is required of users | Automated ECG analysis p17 |
|  | 27c | Discuss any next steps for future research, with a specific view to applicability and generalizability of the model | P17 |

**Supplementary Table 1. Congenital Heart Disease Subgroups and Representative Lesions**

| CHD Group | Representative Lesions |
| --- | --- |
| Right Heart (RH)/Cono-truncal Lesions | Tetralogy of Fallot (TOF), Ebstein anomaly, Valvar Pulmonary Stenosis (VPS), Pulmonary Atresia with Intact Ventricular Septum (PAIVS), Double-Chambered Right Ventricle (DCRV), Truncus Arteriosus, Transposition of the Great Arteries - Arterial Switch Operation (TGA-ASO), TGA with Rastelli, TGA with Double Outlet Right Ventricle (DORV), S/P Rastelli |
| Left Heart (LH) Lesions | Coarctation of the Aorta (COA), Mitral Stenosis (MS), Subaortic Stenosis (SUB-AS), Aortic Stenosis (AS), Supravalvar Aortic Stenosis (SUPRA-AS) |
| Shunt Lesions | Partial Anomalous Pulmonary Venous Return (PAPVR), Atrial Septal Defect (ASD), Ventricular Septal Defect (VSD), Atrioventricular Canal (AV Canal) |
| Cyanotic Heart Disease | Eisenmenger Syndrome (ES), Unrepaired or Palliated Single Ventricle Physiology |
| Other Lesions | Patent Ductus Arteriosus (PDA), Cor Triatriatum |
| Systemic Right Ventricle | Congenitally Corrected Transposition of the Great Arteries (CCTGA), TGA post Mustard procedure |
| Single Ventricle | Fontan Circulation |

**Supplementary Table 2A. Pairwise post-hoc comparisons of age at first visit across AI-ECG diastolic grades (Dunn–Holm)**

| Variable | Comparison | Dunn Z | Dunn p (Holm) |
| --- | --- | --- | --- |
| Age at first visit | G0 vs G1 | −10.867 | <0.001 |
|  | G0 vs G2 | −10.186 | <0.001 |
|  | G1 vs G2 | 5.217 | <0.001 |
|  | G0 vs G3 | −12.130 | <0.001 |
|  | G1 vs G3 | 2.438 | 0.0148 |
|  | G2 vs G3 | −3.631 | <0.001 |

Post-hoc results following a significant Kruskal–Wallis omnibus test (Table 1). Entries are Dunn’s test Z and Holm-adjusted p-values for all grade-to-grade comparisons. *G = grade.* Two-sided α=0.05.

**Supplementary Table 2A. Categorical variables-pairwise comparisons across AI-ECG grades**

| Variable | Comparison | OR (event=1) | 95% CI | Holm p |
| --- | --- | --- | --- | --- |
| Atrial arrhythmia | G2 vs G0 | 2.27 | 1.98–2.62 | <0.001 |
|  | G3 vs G0 | 5.67 | 4.79–6.72 | <0.001 |
|  | G2 vs G1 | 2.59 | 1.84–3.69 | <0.001 |
|  | G3 vs G1 | 6.44 | 4.53–9.32 | <0.001 |
|  | G3 vs G2 | 2.49 | 2.06–3.02 | <0.001 |
| Atrial fibrillation | G2 vs G0 | 2.29 | 1.95–2.70 | <0.001 |
|  | G3 vs G0 | 6.07 | 5.08–7.26 | <0.001 |
|  | G2 vs G1 | 2.73 | 1.80–4.28 | <0.001 |
|  | G3 vs G1 | 7.22 | 4.73–11.39 | <0.001 |
|  | G3 vs G2 | 2.65 | 2.17–3.24 | <0.001 |
| Atrial flutter/tachycardia | G2 vs G0 | 1.90 | 1.59–2.28 | <0.001 |
|  | G3 vs G0 | 3.37 | 2.76–4.11 | <0.001 |
|  | G2 vs G1 | 2.05 | 1.33–3.27 | 0.0012 |
|  | G3 vs G1 | 3.63 | 2.33–5.85 | <0.001 |
|  | G3 vs G2 | 1.77 | 1.41–2.22 | <0.001 |
| Ventricular arrhythmia | G2 vs G0 | 1.87 | 1.45–2.40 | <0.001 |
|  | G3 vs G0 | 2.94 | 2.22–3.86 | <0.001 |
|  | G2 vs G1 | 2.02 | 1.09–4.08 | 0.048 |
|  | G3 vs G1 | 3.17 | 1.69–6.48 | <0.001 |
|  | G3 vs G2 | 1.57 | 1.15–2.15 | 0.012 |
| Sustained VT | G3 vs G0 | 4.24 | 2.76–6.44 | <0.001 |
|  | G3 vs G1 | 8.76 | 2.25–75.13 | <0.001 |
|  | G3 vs G2 | 2.69 | 1.62–4.53 | <0.001 |
| Non-sustained VT | G2 vs G0 | 1.80 | 1.32–2.45 | <0.001 |
|  | G3 vs G0 | 2.75 | 1.95–3.84 | <0.001 |
|  | G3 vs G1 | 2.60 | 1.25–6.07 | 0.025 |
| CKD | G1 vs G0 | 2.14 | 1.19–3.62 | 0.014 |
|  | G2 vs G0 | 2.80 | 2.11–3.70 | <0.001 |
|  | G3 vs G0 | 4.63 | 3.43–6.23 | <0.001 |
|  | G3 vs G1 | 2.17 | 1.25–3.97 | 0.011 |
|  | G3 vs G2 | 1.65 | 1.20–2.27 | 0.006 |
| OSA | G0 vs G1 | 1.61 | 1.14–2.24 | 0.021 |
|  | G2 vs G0 | 1.36 | 1.13–1.64 | 0.006 |
|  | G3 vs G0 | 1.42 | 1.12–1.78 | 0.018 |
| CAD | G1 vs G0 | 2.47 | 1.51–3.89 | 0.001 |
|  | G2 vs G0 | 2.21 | 1.68–2.88 | <0.001 |
|  | G3 vs G0 | 2.01 | 1.41–2.82 | <0.001 |
| Stroke | G1 vs G0 | 2.74 | 1.71–4.27 | <0.001 |
|  | G2 vs G0 | 1.84 | 1.37–2.44 | <0.001 |
|  | G3 vs G0 | 2.23 | 1.59–3.11 | <0.001 |
| Endocarditis | G2 vs G0 | 2.28 | 1.57–3.30 | <0.001 |
|  | G3 vs G0 | 3.63 | 2.43–5.36 | <0.001 |
| Cirrhosis | G1 vs G3 | 3.64 | 1.54–10.49 | 0.005 |
|  | G2 vs G0 | 5.57 | 3.55–8.87 | <0.001 |
|  | G3 vs G0 | 9.97 | 6.28–16.01 | <0.001 |
|  | G3 vs G2 | 1.79 | 1.18–2.71 | 0.013 |
| Venous thrombosis | G2 vs G0 | 2.14 | 1.34–3.39 | 0.005 |
|  | G3 vs G0 | 2.76 | 1.60–4.61 | <0.001 |
| Obesity | G0 vs G1 | 1.64 | 1.26–2.13 | 0.001 |
|  | G1 vs G2 | 0.60 | 0.45–0.81 | 0.0019 |
|  | G1 vs G3 | 0.46 | 0.34–0.64 | <0.001 |
|  | G2 vs G3 | 0.77 | 0.61–0.97 | 0.0517 |

Post-hoc analysis categorical variables with a significant global test in Table 1, pairwise 2×2 comparisons between grades are shown as OR (event=1) with 95% confidence intervals and Holm-adjusted p-values (α=0.05). OR>1 indicates greater prevalence in the first group listed. Abbreviations: VT, ventricular tachycardia; VF, ventricular fibrillation; OSA, obstructive sleep apnea; CKD, chronic kidney disease; CAD, coronary artery disease.

**Supplementary Table 3.** Correlation between AI-ECG Estimated Filling Pressures and Invasive Hemodynamic Parameters

| Variable | Overall | RH/Cono-truncal lesions | Left heart disease | Shunt lesion | Cyanotic HD | Other CHD | Systemic RV | Fontan palliation |
| --- | --- | --- | --- | --- | --- | --- | --- | --- |
| RA Pressure (mmHg) | 0.43 (0.39-0.47), n=1715 | 0.30 (0.22-0.37), n=560 | 0.46 (0.33-0.57), n=205 | 0.45 (0.38-0.52), n=556 | 0.11 (-0.17-0.34), n=63 | 0.62 (-1.00-1.00), n=5 | 0.18 (-0.01-0.37), n=110 | 0.26 (0.11-0.39), n=216 |
| RV Systolic Pressure (mmHg) | 0.43 (0.39-0.47), n=1688 | 0.14 (0.06-0.21), n=604 | 0.64 (0.55-0.71), n=211 | 0.53 (0.47-0.59), n=638 | -0.06 (-0.30-0.23), n=63 | 0.69 (0.24-0.89), n=29 | -0.02 (-0.17-0.15), n=126 | 0.66 (0.28-0.85), n=17 |
| RV End-Diastolic Pressure (mmHg) | 0.29 (0.25-0.34), n=1691 | 0.21 (0.13-0.29), n=602 | 0.37 (0.24-0.48), n=211 | 0.33 (0.26-0.40), n=641 | 0.10 (-0.16-0.34), n=63 | 0.45 (0.03-0.74), n=33 | 0.04 (-0.15-0.22), n=123 | 0.44 (-0.19-0.83), n=18 |
| PA Systolic Pressure (mmHg) | 0.36 (0.32-0.41), n=1665 | 0.26 (0.18-0.33), n=565 | 0.66 (0.56-0.73), n=206 | 0.54 (0.48-0.60), n=584 | -0.15 (-0.35-0.07), n=70 | 0.82 (0.64-0.92), n=34 | 0.17 (-0.02-0.35), n=130 | 0.29 (0.06-0.48), n=76 |
| PA Diastolic Pressure (mmHg) | 0.41 (0.37-0.45), n=1661 | 0.26 (0.19-0.34), n=567 | 0.60 (0.50-0.68), n=204 | 0.49 (0.42-0.55), n=582 | -0.04 (-0.28-0.21), n=69 | 0.73 (0.52-0.85), n=34 | 0.18 (-0.02-0.34), n=129 | 0.45 (0.26-0.61), n=76 |
| PA Mean Pressure (mmHg) | 0.30 (0.26-0.34), n=1815 | 0.30 (0.22-0.38), n=566 | 0.67 (0.57-0.74), n=202 | 0.52 (0.45-0.58), n=579 | -0.13 (-0.33-0.10), n=72 | 0.59 (0.22-0.82), n=34 | 0.21 (0.03-0.39), n=130 | 0.31 (0.19-0.43), n=232 |
| Pulmonary Artery Wedge Pressure (mmHg) | 0.33 (0.28-0.38), n=1460 | 0.22 (0.14-0.32), n=464 | 0.56 (0.44-0.66), n=177 | 0.45 (0.36-0.53), n=441 | 0.24 (-0.00-0.48), n=56 | 0.40 (-1.00-1.00), n=5 | 0.21 (-0.01-0.39), n=103 | 0.27 (0.13-0.38), n=214 |
| LV End-Diastolic Pressure (mmHg) | 0.09 (0.03-0.15), n=1124 | 0.13 (0.03-0.24), n=344 | 0.34 (0.20-0.47), n=155 | 0.18 (0.07-0.28), n=354 | 0.15 (-0.12-0.41), n=43 | 0.15 (-0.39-0.61), n=20 | 0.18 (-0.07-0.39), n=87 | 0.11 (-0.07-0.28), n=121 |
| Systolic Blood Pressure (mmHg) | -0.01 (-0.05-0.05), n=1637 | 0.10 (0.02-0.18), n=572 | 0.14 (0.04-0.25), n=319 | -0.02 (-0.13-0.08), n=364 | -0.08 (-0.36-0.22), n=50 | 0.62 (-0.59-1.00), n=8 | -0.04 (-0.21-0.13), n=128 | 0.03 (-0.11-0.18), n=196 |
| Diastolic Blood Pressure (mmHg) | -0.11 (-0.16--0.06), n=1630 | -0.02 (-0.10-0.06), n=569 | -0.02 (-0.13-0.09), n=318 | -0.14 (-0.23--0.04), n=361 | -0.19 (-0.45-0.08), n=50 | -0.51 (-1.00-0.39), n=8 | -0.16 (-0.32-0.02), n=128 | -0.02 (-0.15-0.13), n=196 |
| Mean Blood Pressure (mmHg) | -0.08 (-0.13--0.04), n=1621 | 0.01 (-0.07-0.09), n=573 | 0.03 (-0.08-0.14), n=315 | -0.07 (-0.18-0.03), n=357 | -0.22 (-0.48-0.06), n=49 | 0.16 (-0.68-0.92), n=8 | -0.18 (-0.34--0.01), n=124 | -0.02 (-0.17-0.14), n=195 |
| Mixed Venous Saturation (%) | -0.36 (-0.41--0.31), n=1188 | -0.33 (-0.42--0.23), n=376 | -0.64 (-0.72--0.52), n=157 | -0.30 (-0.38--0.21), n=450 | 0.08 (-0.28-0.37), n=46 | -0.40 (-1.00-1.00), n=4 | -0.11 (-0.31-0.10), n=98 | -0.27 (-0.50-0.00), n=57 |
| Pulmonary Artery Saturation (%) | -0.39 (-0.43--0.35), n=1650 | -0.30 (-0.38--0.21), n=454 | -0.59 (-0.67--0.49), n=183 | -0.33 (-0.40--0.25), n=588 | 0.07 (-0.18-0.32), n=66 | -0.41 (-0.72-0.03), n=27 | -0.02 (-0.23-0.17), n=112 | -0.25 (-0.37--0.13), n=220 |
| Pulmonary Vein Saturation (%) | -0.26 (-0.33--0.18), n=640 | -0.15 (-0.38-0.06), n=80 | 0.09 (-0.26-0.40), n=35 | -0.23 (-0.32--0.13), n=372 | -0.21 (-0.49-0.12), n=48 | -1.00 (-1.00--1.00), n=4 | -0.15 (-0.40-0.13), n=47 | 0.04 (-0.29-0.32), n=54 |
| Aortic Saturation (%) | -0.39 (-0.44--0.35), n=1454 | -0.25 (-0.35--0.16), n=407 | -0.42 (-0.53--0.30), n=198 | -0.39 (-0.47--0.31), n=482 | -0.24 (-0.49-0.01), n=55 | -0.84 (-1.00--0.45), n=6 | -0.01 (-0.23-0.19), n=84 | -0.26 (-0.37--0.13), n=222 |
| Qs Systemic Flow Index (L/min/m²) | -0.18 (-0.23--0.12), n=1168 | -0.24 (-0.32--0.13), n=383 | -0.32 (-0.46--0.16), n=140 | -0.15 (-0.26--0.04), n=297 | 0.07 (-0.21-0.36), n=50 | NA, n=1 | -0.12 (-0.34-0.12), n=86 | -0.02 (-0.15-0.13), n=211 |
| PVR Index (mmHg/(L/min/m²)) | 0.18 (0.11-0.24), n=942 | 0.22 (0.10-0.34), n=239 | 0.49 (0.33-0.62), n=126 | 0.41 (0.30-0.52), n=227 | 0.06 (-0.25-0.37), n=45 | 1.00 (1.00-1.00), n=4 | 0.19 (-0.04-0.40), n=91 | 0.16 (0.02-0.29), n=210 |
| SVR Index (mmHg/(L/min/m²)) | -0.00 (-0.09-0.08), n=550 | 0.03 (-0.13-0.18), n=145 | 0.25 (-0.02-0.46), n=69 | 0.10 (-0.08-0.27), n=142 | -0.22 (-0.64-0.25), n=19 | 0.60 (-1.00-1.00), n=4 | 0.09 (-0.21-0.39), n=51 | -0.05 (-0.22-0.14), n=120 |

Correlation between AI-ECG Estimated Filling Pressures and Invasive Hemodynamic Parameters in the overall cohort and by CHD subgroups. Strength of correlation is indicated by Spearman’s correlation coefficients.

**Supplementary Table 4.** Diagnostic performance of AI‑ECG–predicted pulmonary artery wedge pressure (PAWP) at candidate hemodynamic cut‑offs (15, 18, 20, 22 mm Hg), n=1460

| PAWP Cut-off (mmHg) | Prevalence (95% CI) | Sensitivity (95% CI) | Specificity (95% CI) | PPV (95% CI) | NPV (95% CI) | Accuracy (95% CI) | AUC (95% CI) | Best Threshold |
| --- | --- | --- | --- | --- | --- | --- | --- | --- |
| 15 | 0.31 (0.29–0.34) | 0.66 (0.62–0.71) | 0.67 (0.64–0.70) | 0.48 (0.44–0.52) | 0.81 (0.79–0.84) | 0.67 (0.64–0.69) | 0.70 (0.67–0.73) | 0.5 |
| 18 | 0.18 (0.17–0.21) | 0.75 (0.70–0.80) | 0.64 (0.61–0.67) | 0.32 (0.28–0.36) | 0.92 (0.90–0.94) | 0.66 (0.63–0.68) | 0.73 (0.69–0.76) | 0.5 |
| 20 | 0.13 (0.12–0.15) | 0.77 (0.70–0.83) | 0.62 (0.60–0.65) | 0.24 (0.21–0.28) | 0.95 (0.93–0.96) | 0.65 (0.62–0.67) | 0.74 (0.70–0.78) | 0.51 |
| 22 | 0.10 (0.08–0.11) | 0.72 (0.64–0.79) | 0.69 (0.67–0.72) | 0.20 (0.17–0.24) | 0.96 (0.94–0.97) | 0.70 (0.67–0.72) | 0.74 (0.70–0.79) | 0.66 |

**
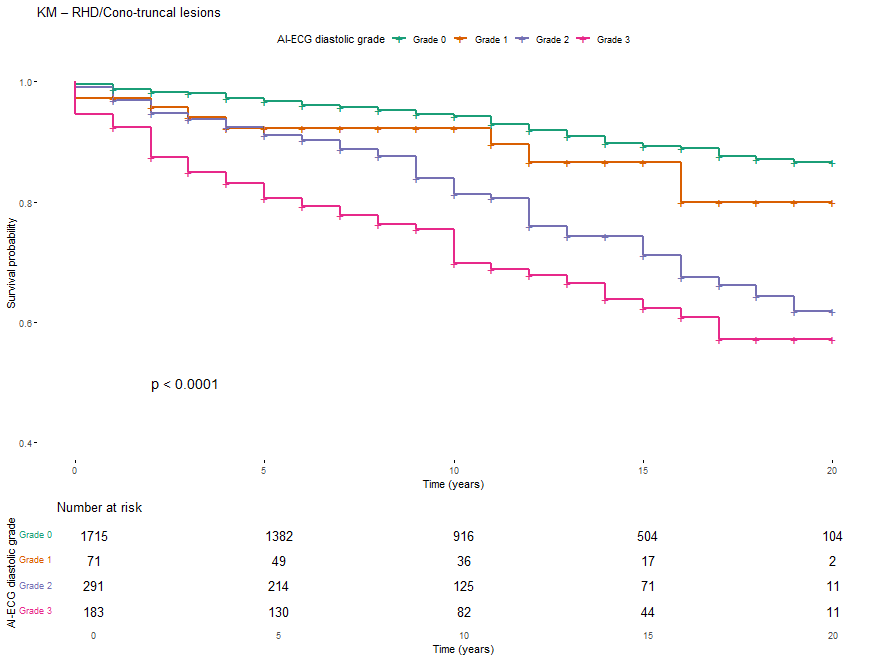
**

**Supplementary Figure 1**: Kaplan-Meier survival curves comparing survival probabilities across AI-ECG diastolic grade in RHD/Cono-truncal lesions. The log-rank test shows a statistically significant difference in survival (p=<0.0001).

**
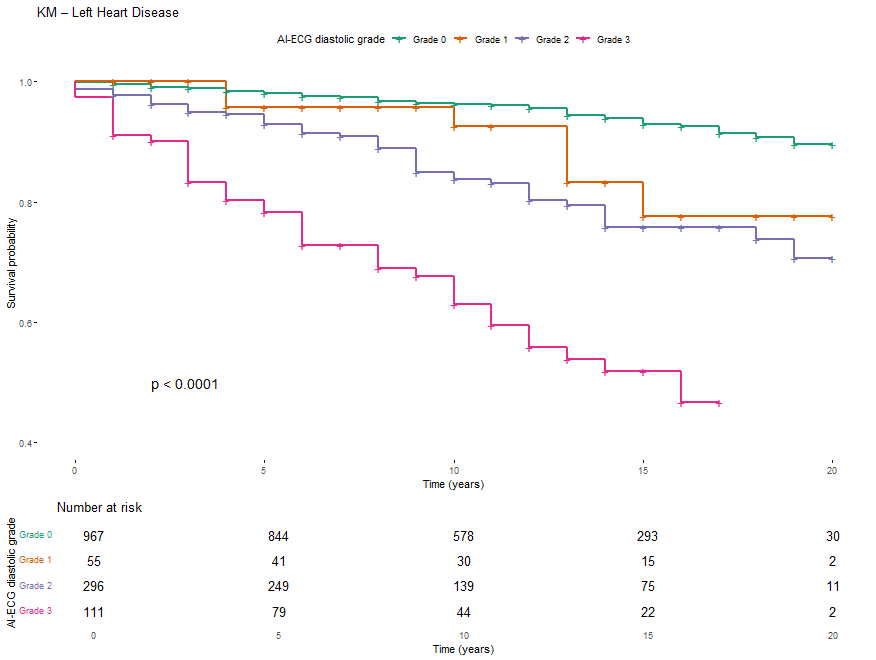
Supplementary Figure 2**: Kaplan-Meier survival curves comparing survival probabilities across AI-ECG estimated diastolic grade in patients with left heart disease. The log-rank test shows a statistically significant difference in survival (p=<0.0001).

**
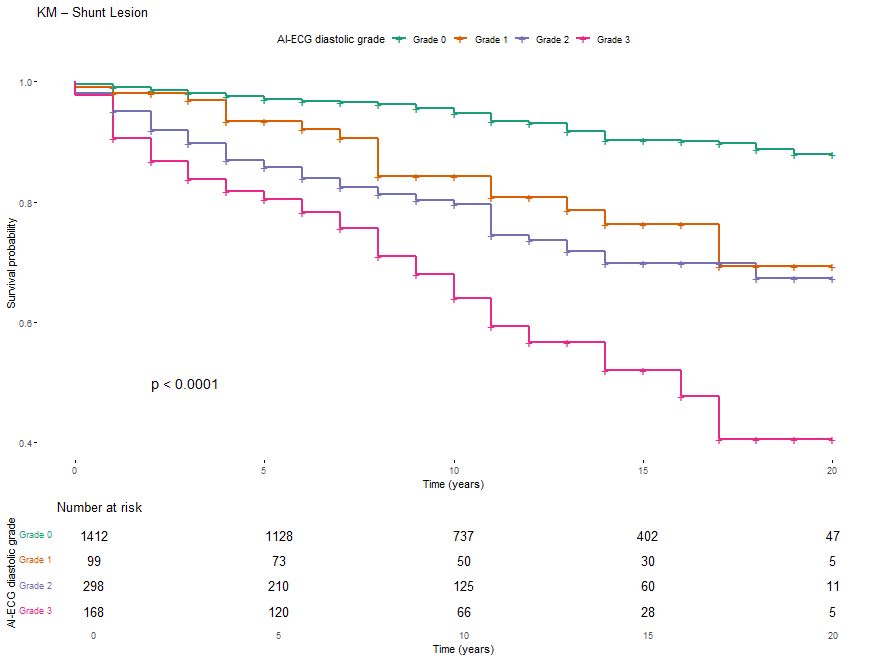
Supplementary Figure 3**: Kaplan-Meier survival curves comparing survival probabilities across AI-ECG estimated diastolic grade in patients with shunt lesions. The log-rank test shows a statistically significant difference in survival (p=<0.0001).

**
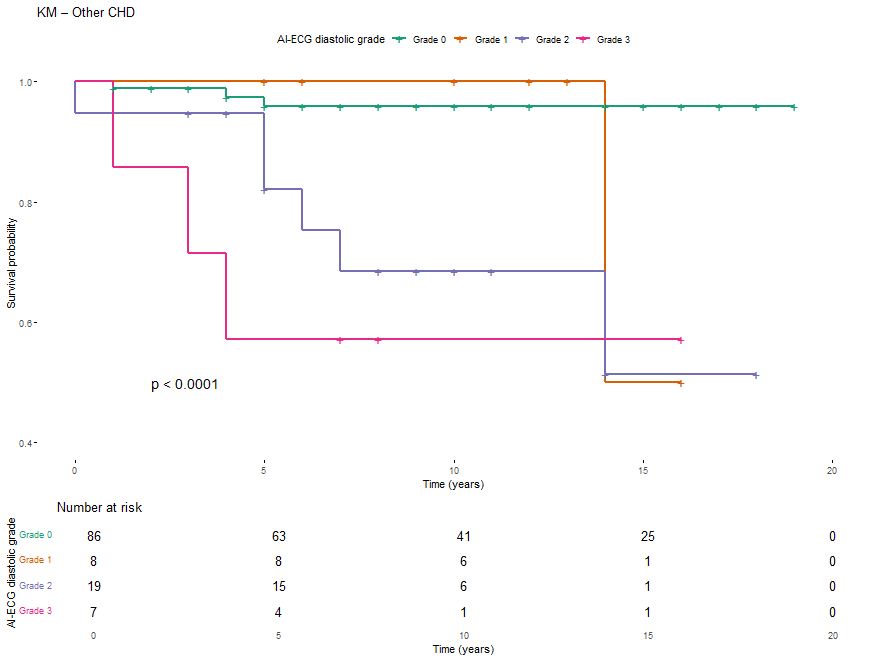
Supplementary Figure 4**: Kaplan-Meier survival curves comparing survival probabilities across AI-ECG diastolic grade in Other CHD. The log-rank test shows a statistically significant difference in survival (p=<0.0001).

**
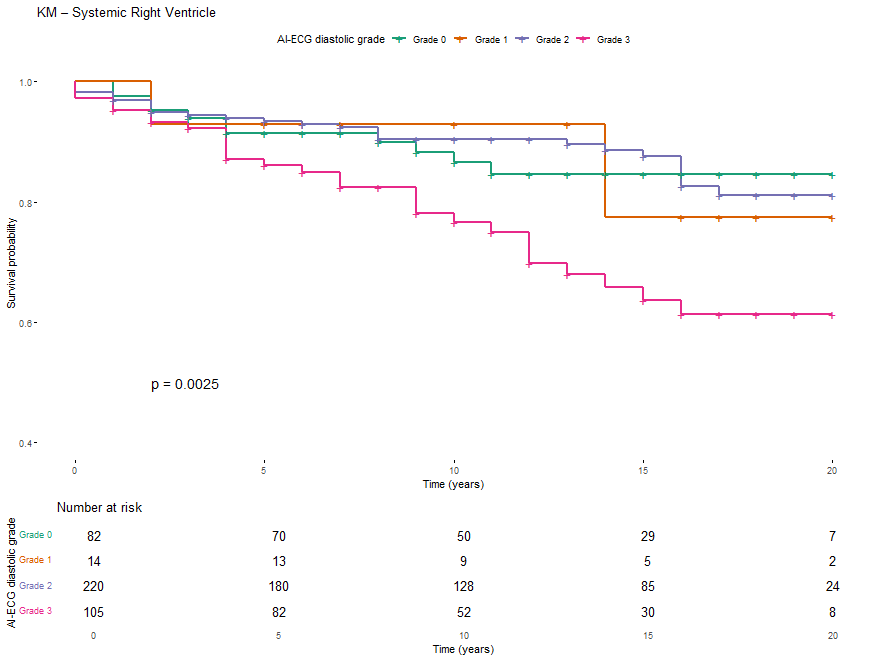
Supplementary Figure 5**: Kaplan-Meier survival curves comparing survival probabilities across AI-ECG diastolic grade in Systemic Right Ventricle. The log-rank test does show statistically significant difference in survival (p=0.0025).

**
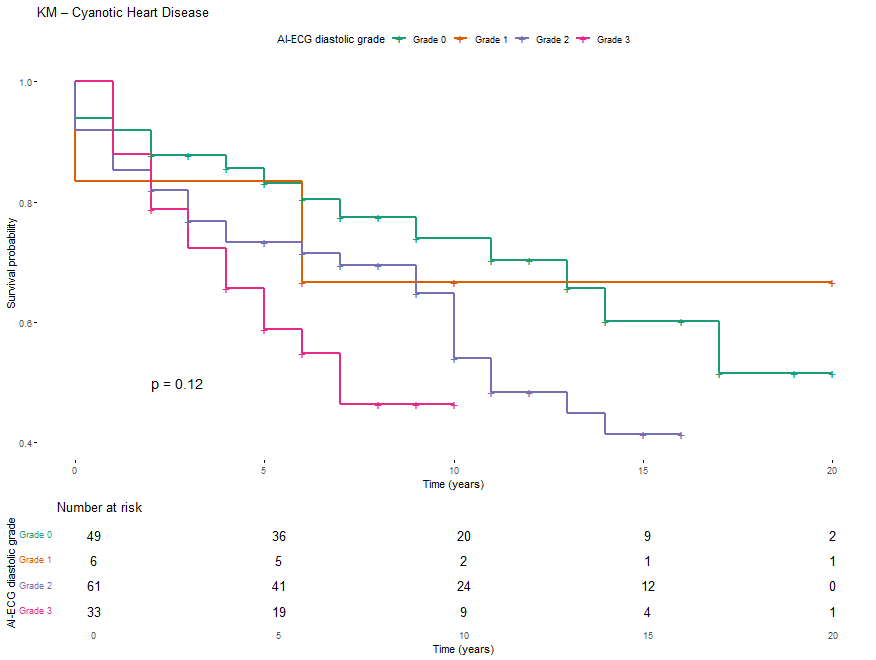
Supplementary Figure 6**: Kaplan-Meier survival curves comparing survival probabilities across AI-ECG estimated diastolic grade in patients with unrepaired cyanotic heart disease. The log-rank test does not show statistically significant difference in survival (p=0.12).

**
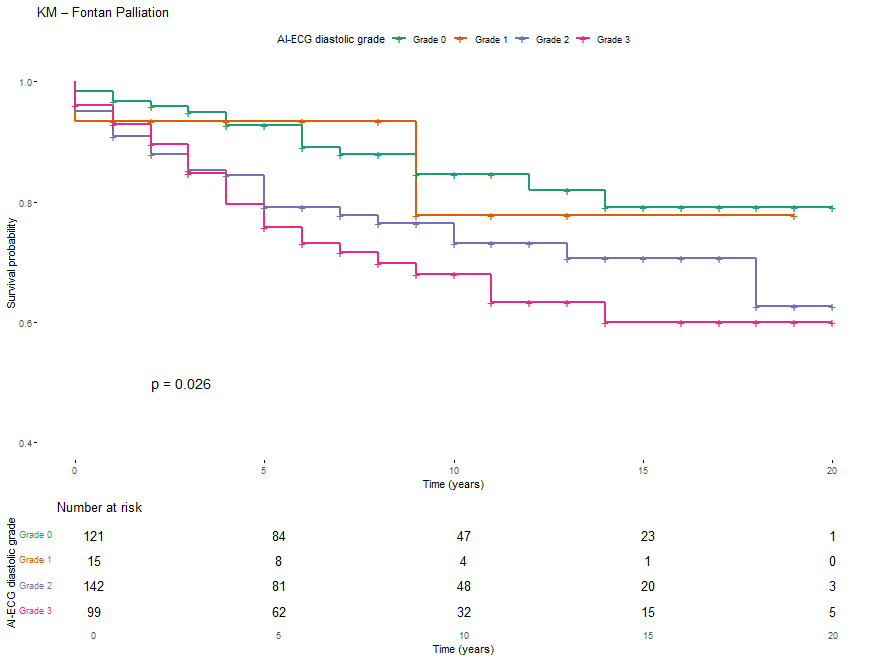
Supplementary Figure 7**: Kaplan-Meier survival curves comparing survival probabilities across AI-ECG estimated diastolic grade in patients with Fontan palliation. The log-rank test does show statistically significant difference in survival (p=0.026).


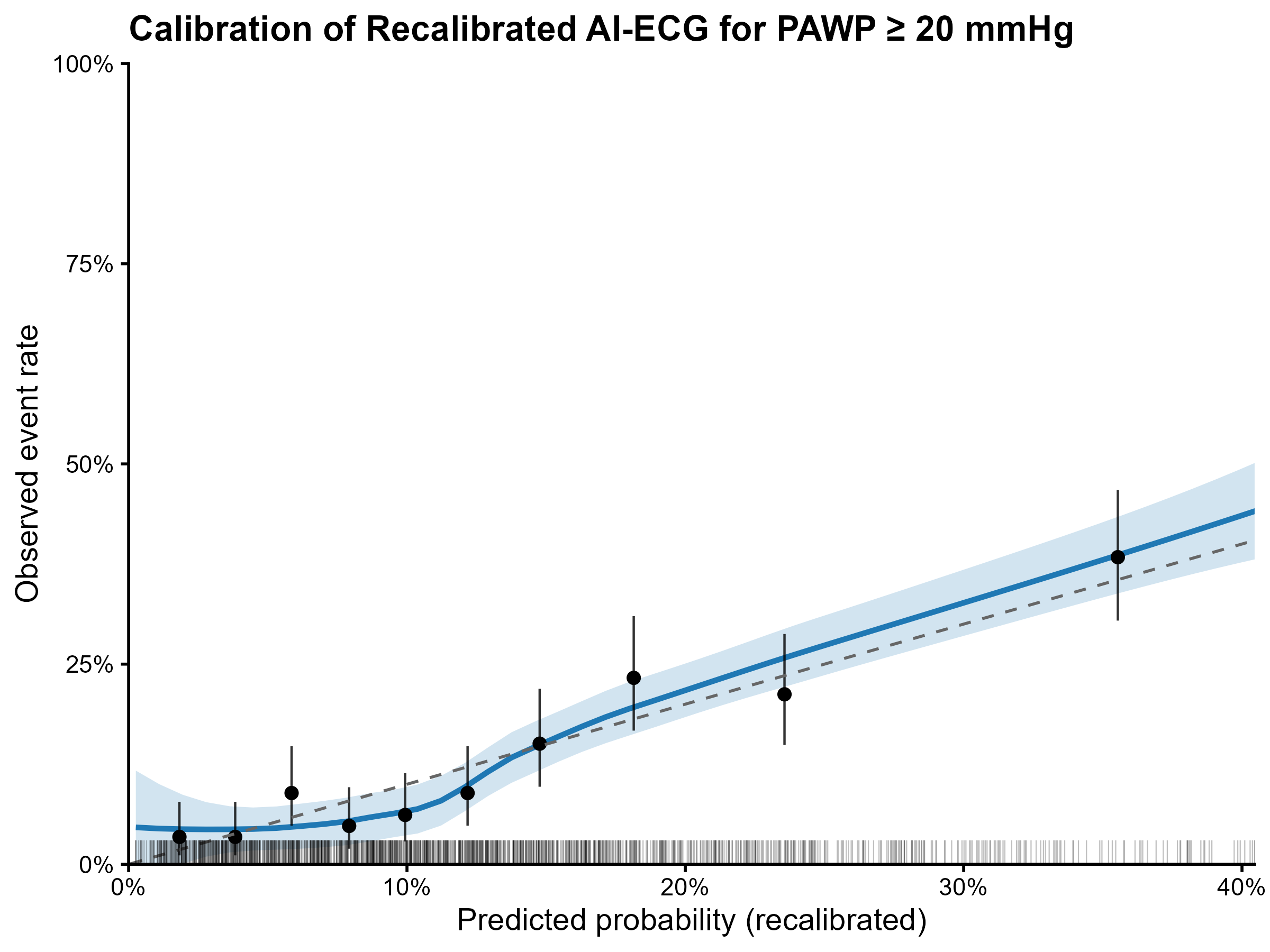


**Supplementary Figure 8**: Calibration curve of the recalibrated AI-ECG model for predicting elevated pulmonary artery wedge pressure >20. The blue line represents the observed event rate across predicted probability values using LOESS smoothing, with the shaded area indicating the 95% confidence interval; black points show decile-wise observed rates. The dashed diagonal line represents perfect calibration (predicted = observed).
